# Single-cell transcriptome profiling reveals heterogeneous neutrophils with prognostic values in sepsis

**DOI:** 10.1016/j.isci.2022.105301

**Published:** 2022-10-07

**Authors:** Yucai Hong, Lin Chen, Jian Sun, Lifeng Xing, Yi Yang, Xiaohong Jin, Huabo Cai, Lianlian Dong, Liping Zhou, Zhongheng Zhang

**Affiliations:** 1 Department of Emergency Medicine, Key Laboratory of Precision Medicine in Diagnosis and Monitoring Research of Zhejiang Province, Sir Run Run Shaw Hospital, Zhejiang University School of Medicine, Hangzhou 310016, China; 2 Department of Critical Care Medicine, Affiliated Jinhua Hospital, Zhejiang University School of Medicine, Jinhua, China; 3 Department of Critical Care Medicine, Lishui Center Hospital, Lishui, Zhejiang 323000, China; 4 Department of Emergency Medicine, Sir Run Run Shaw Hospital, Zhejiang University School of Medicine, Hangzhou 310016, China; 5 Department of Emergency Medicine, The Second Hospital of Jiaxing, Jiaxing, 314000, P.R.China; 6 Department of Emergency Medicine, The Affiliated Wenling Hospital of Wenzhou Medical University, Wenling 317500, Zhejiang Province, China; 7 Key Laboratory of Digital Technology in Medical Diagnostics Of Zhejiang Province, Hangzhou, Zhejiang, China

**Keywords:** Biological sciences, Immunology, Transcriptomics

## Abstract

Neutrophils constitute the largest proportion of nucleated peripheral blood cells, and neutrophils have substantial heterogeneity. We profiled nearly 300,000 human peripheral blood cells in this study using single-cell RNA sequencing. A large proportion (>50%) of these cells were annotated as neutrophils. Neutrophils were further clustered into four subtypes, including Neu1, Neu2, Neu3, and Neu4. Neu1 is characterized by high expression of MMP9, HP, and RGL4. Neu1 was associated with septic shock and significantly correlated with the sequential organ failure assessment (SOFA) score. A gene expression module in Neu1 named Neu1_C (characterized by expression of NFKBIA, CXCL8, G0S2, and FTH1) was highly predictive of septic shock with an area under the curve of 0.81. The results were extensively validated in external bulk datasets by using single-cell deconvolution methods. In summary, our study establishes a general framework for studying neutrophil-related mechanisms, prognostic biomarkers, and potential therapeutic targets for septic shock.

## Highlights

• Neutrophils were clustered into four subtypes, including Neu1, Neu2, Neu3, and Neu4
• Neu1 was associated with septic shock
• Neu1 was correlated with the sequential organ failure assessment (SOFA) score
• A gene expression module in Neu1 named Neu1_C was highly predictive of septic shock

## Graphical abstract

**Figure:**
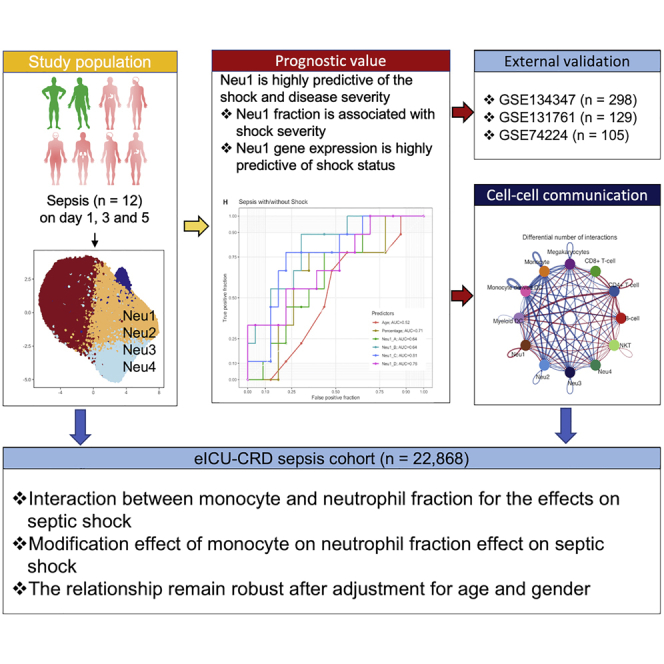

## Introduction

Sepsis is a leading cause of morbidity and mortality in the intensive care unit (ICU). The stages of sepsis include sepsis, severe sepsis, and septic shock (Evans et al., 2021). Septic shock is the most severe form of sepsis, with an estimated mortality rate of 30–50% (Bauer et al., 2020; Ma et al., 2021; Yu et al., 2021). However, the pathogenesis of septic shock is not well established. Neutrophils account for the largest proportion of nucleated peripheral blood cells, which migrate from circulating blood to infected tissues in response to inflammatory stimuli. Neutrophils have been found to have an immunomodulatory capacity in many disease conditions (Hassani et al., 2020; Tay et al., 2020). Neutrophils are greatly expanded and enriched during sepsis response, playing important roles in the pathogenesis and development of sepsis (Darcy et al., 2014; Leite et al., 2021; Takizawa et al., 2021; Uhel et al., 2017). Neutrophils are heterogeneous, with each subtype having distinct transcriptome profiles and biological functions at different severity stages (Kangelaris et al., 2021; Seman and Robinson, 2021). However, the heterogeneity of neutrophils and related clinical implications are not fully explored. Xie et al. comprehensively described the transcriptional landscape of neutrophil maturation, function and fate decision, and eight neutrophil populations were defined by distinct molecular signatures (Xie et al., 2020).

Single-cell RNA sequencing (scRNA-seq) is a powerful tool for the exploration of heterogeneity at the single-cell level. Here, we adopt an unbiased approach to explore the heterogeneity of human peripheral blood cells. Specifically, we focus on the heterogeneity of peripheral blood neutrophils, as well as the underlying mechanisms driving the development of subtypes. The association of neutrophil subtype abundance with shock status is explored and tested in an independent dataset. The study provides a comprehensive reference map of neutrophil transcriptional states in sepsis and septic shock patients.

## Results

### Human peripheral cell atlas in sepsis

**Figure 1:**
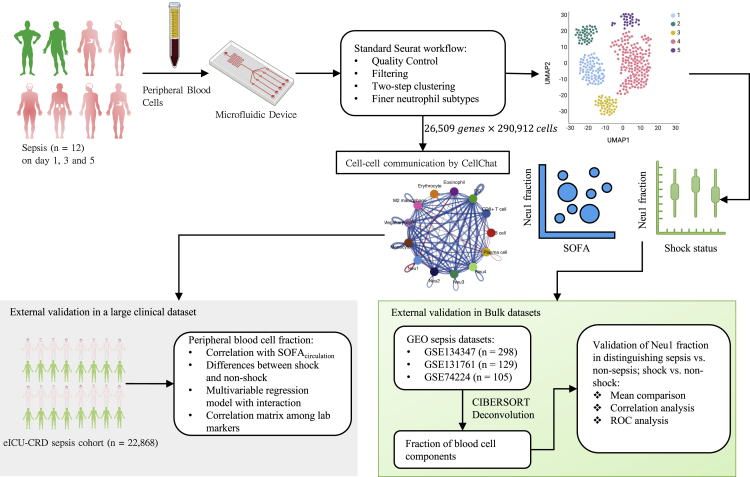
Workflow of the analysis (A) total of 32 peripheral blood samples were collected from 12 sepsis patients (i.e., four patients were discharged or died before day 5 and thus they had missing samples on day 5). Single-cell RNA sequencing (scRNA-Seq) was performed by using the microfluidic technique. Standard Seurat workflow was applied for cell clustering and annotation. A two-step clustering was applied to the neutrophils, and four subtypes of neutrophils were identified. The fraction of neutrophil subtypes was correlated with shock status and severity score. The results were extensively validated in an external dataset, with cell type fractions obtained via deconvolution methods. Cell-cell communications were explored using the CellChat workflow.

**Figure 2:**
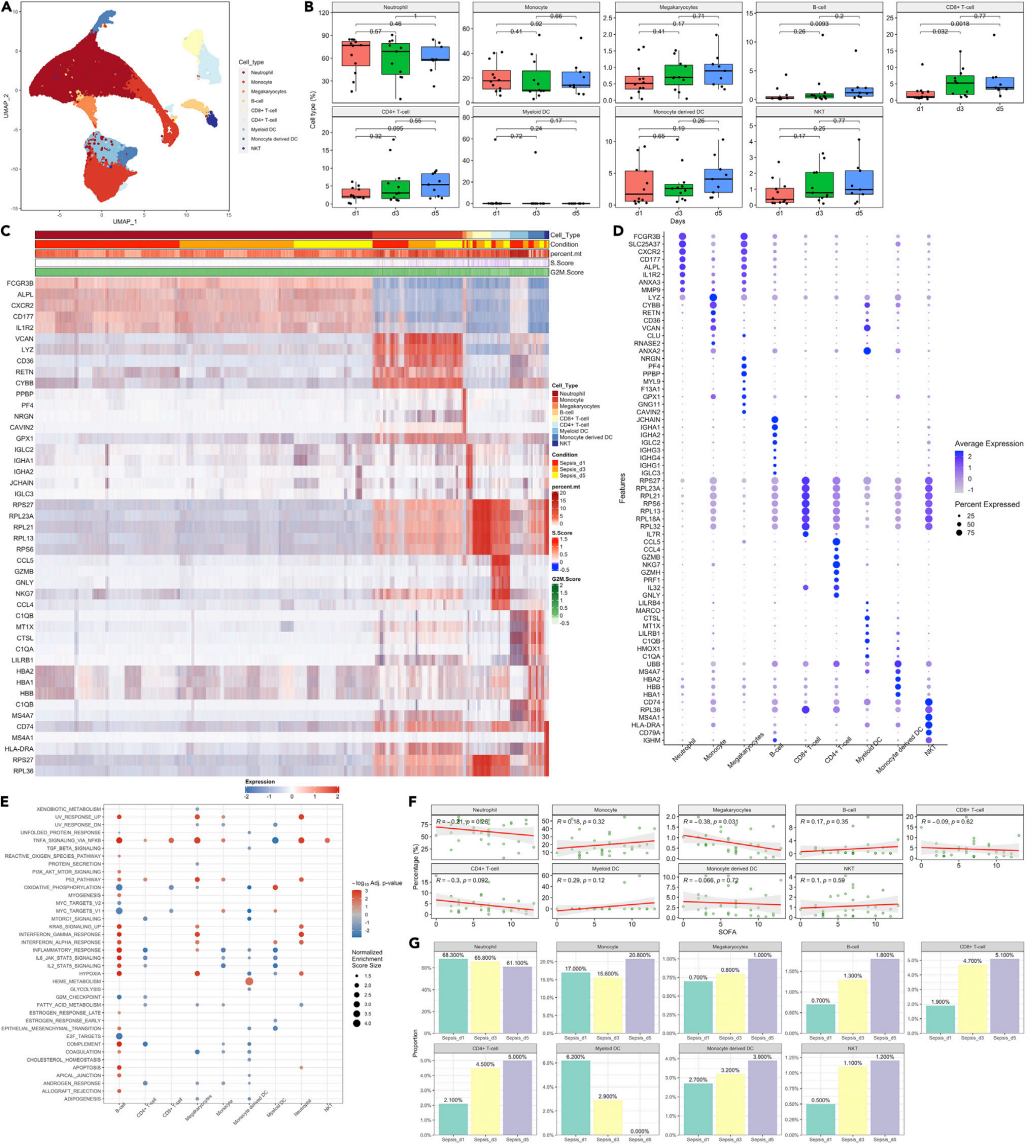
Raw clustering of major cell types (A) distribution of blood cells in the two-dimension UMAP space. Different colors indicate different cell types. (B) Changes of cell type fraction over days 1, 3, and 5. No significant difference is observed across the disease course. Data are represented with boxplot indicating the median, and interquartile range. Differences between days are tested using Student’s t-test and the p values were indicated in the figure for each comparison. (C) Heatmap showing the markers genes for each cell type. (D) Dot plot showing the marker genes for each cell type. The size of the circle represents the percentage of cells expressing relevant genes. (E) GO enrichment analysis for marker genes in each cell type comparing mild versus severe sepsis. The negative value indicates downregulated pathways. (F) Correlation between SOFA and the fraction of cell types. (G) Distribution of cell type fractions across days 1, 3, and 5.

A total of 32 peripheral blood samples from 12 sepsis patients on days 1, 3, and 5 were obtained for scRNA-seq profiling (i.e., Four patients were either discharged or died before day 5 and thus their samples were missing on day 5, Figure 1). After rigorous quality control (Figure S1), we obtained 290,912 high-quality cells with an average of 880 genes (2627 UMIs) per cell, resulting in a total of 26,509 unique genes being detected in all cells. Unbiased, graph-based clustering uniform manifold approximation and projection (UMAP) identified 9 major cell populations (Figure 2A and S3) (Becht et al., 2018). Notably, the neutrophils accounted for most of the cell population. There was no significant difference in the fraction of neutrophils during sepsis on days 1 through 5 (Figure 2B). Marker genes of the neutrophil cluster included FCGR3B, CXCR2, IL1R2, CD177, MMP9, and S100A9 (Figures 2C and 2D and Table S1), which were consistent with that reported in the PanglaoDB (Franzén et al., 2019). In comparison to mild sepsis (SOFA <10), genes in neutrophils were significantly enriched in biological pathways such as TNF-α signaling via NFKB, Interferon-γ, and Interferon-α response (Figure 2E). The enrichment pathways comparing severe versus mild sepsis were generally consistent across the disease course on days 1, 3 and 5 (Figure S4). The component cells were not significantly associated with the SOFA score, except for the megakaryocytes, whose fraction was negatively associated with the SOFA score (R = −0.38; p = 0.031; Figure 2F). CD4^+^ T-cell was reduced during the acute phase of sepsis, and then gradually increased during recovery (Figure 2G). The inferred neutrophil proportion correlated well with the measured proportion (R_Pearson_ = 0.63; p < 0.01; Figure S5).

### Subtypes of neutrophils

**Figure 3:**
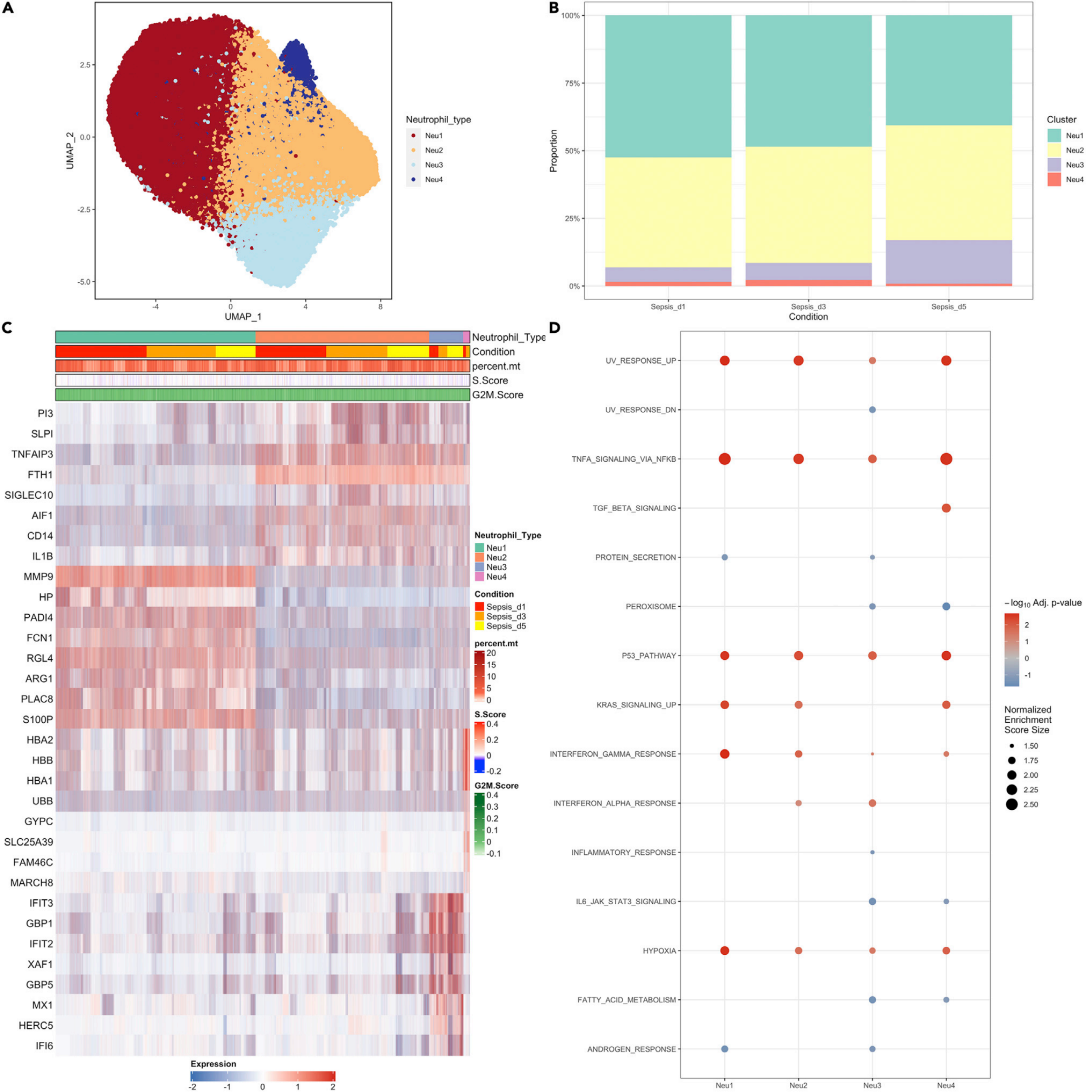
The second clustering of neutrophils (A) distribution of neutrophil types, namely, Neu1, Neu2, Neu3, and Neu4 in the two-dimension UMAP space. (B) Proportion of neutrophil types across sepsis course. (C) Heatmap showing marker genes for each neutrophil type. (D) Enrichment analysis for pathways associated with each neutrophil subtype, by comparing severe versus mild sepsis.

To obtain a stable clustering of neutrophils, we resample the neutrophils and calculate the rand index for concordance of cluster membership at different resolutions. A resolution associated with a significant drop in the Rand index indicated unstable clustering with higher resolution values. Four subtypes of neutrophils were identified: Neu1, Neu2, Neu3, and Neu4 (Figure 3A). Neu1 was expanded in the acute phase of sepsis on day 1, which then gradually declined during the recovery phase (Figure 3B). However, the Neu1 fraction on sepsis day 5 was still higher than the normal control subjects (Figure S6). As compared with other neutrophil subtypes, Neu1 was featured by the expression of MMP9, HP, RGL4, and FCN1. Feature genes of Neu2 included FTH1, TNFAIP3, SIGLEC10, and CD14 (Figures 3C and 3D). The gene OLFM4 was found to be more highly expressed in the Neu1 subtype (Figure S7), which was in line with the finding that OLFM4 expressed in neutrophils was an important biomarker of disease severity in various inflammatory diseases (Kangelaris et al., 2021; Kassam et al., 2021; Liu and Rodgers, 2022). Some biological pathways were more markedly up-regulated (severe versus mild sepsis) in Neu1 than that in other neutrophil types such as hypoxia, interferon-γ response, and TNF-α signaling via NFκB (Figure 3E). Neu1 and Myeloid-Derived Suppressor Cells (MDSC) share some transcriptomic/cell surface biomarkers such as MMP4, CD123 and CD38 (Karakasheva et al., 2018, p. 38; Lee et al., 2018; Shi et al., 2008, p. 123). Cell surface protein was imputed by deep neural networks; some surface markers such as CD123, CD38 and CD69 were more highly expressed in Neu1 than other subtypes (Figure S8). Neu1 count was found to be correlated with the neutrophil count (R_Pearson_ = 0.56; p < 0.01), as well as the band count (R_Pearson_ = 0.60; p < 0.01; Figure S9).

**Figure 4:**
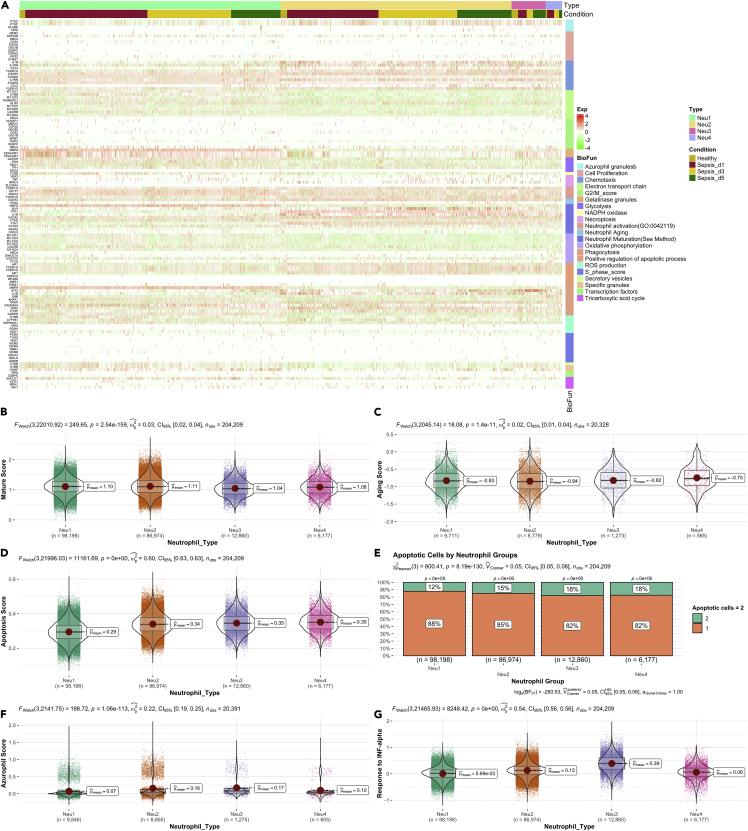
Biological functions of neutrophil subtypes (A–G) Heatmap showing expression of genes associated with prespecified biological functions. Comparisons of (B) mature, (C) aging, (D) apoptosis scores, (E) finite mixture modeling derived apoptotic cells, (F) azurophil, and (G) response to INF-α for the four subtypes of neutrophils. Data are represented with violin and box plots indicating the median, and interquartile range.

Conventional neutrophil biological functions were explored by examining the transcriptional status of genes enriched in relevant biological functions (Xie et al., 2020). Neu1 showed the activated function of gelatinase granules (Figure 4A). Neu1 showed the lowest aging and mature scores than other subtypes (Figures 4B and 4C). Apoptosis score was significantly lower in Neu1 versus other neutrophil subtypes, and fewer apoptotic cells were found in the Neu1 population (Figures 4D and 4E). Neu1 showed reduced response to INF-α. Neutrophils contain azurophils loaded with a wide variety of anti-microbial defensins such as myeloperoxidase, phospholipase A2, acid hydrolases, elastase, and defensins. Neu1 showed reduced azurophils (Figures 4F and 4G) and lower mitochondrial gene percent (Figure S10) as compared with other neutrophil types. NETosis is a program for the formation of neutrophil extracellular traps (NETs), which consist of modified chromatin decorated with bactericidal proteins from granules and cytoplasm. Cathepsin family proteins are important components of the NET (Yang et al., 2016), and their genes (CSTD, CTSH) were upregulated in the Neu1 as compared to other neutrophil subtypes (Table S2). Collectively, these results indicated that Neu1 is the premature type of neutrophil, and its expansion in peripheral blood might indicate a more severe illness that will be addressed in the following sections.

### Neu1 as a biomarker for the severity of sepsis

**Figure 5:**
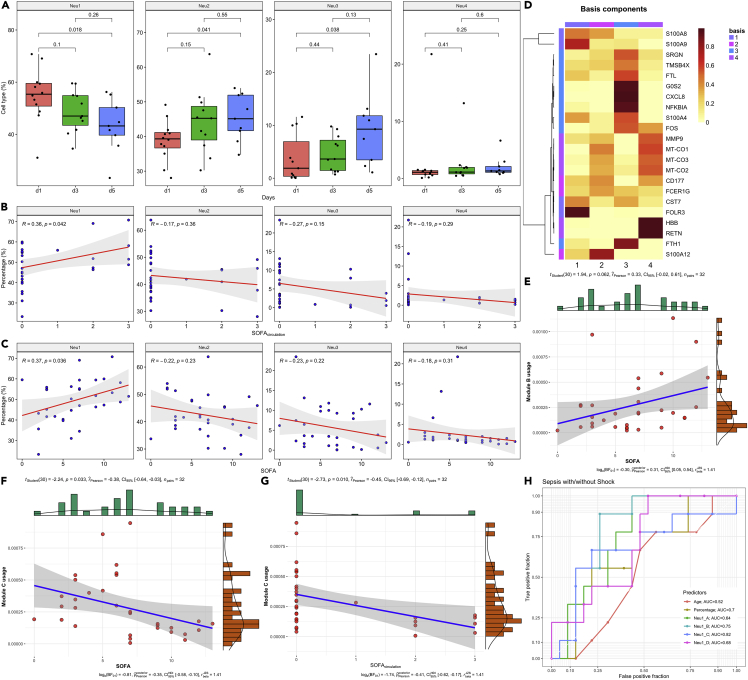
Association of neutrophil subtype fraction and shock status (A) changes in the fraction of neutrophil subtypes from day 1 to day 5. Data are represented with boxplot indicating the median, and interquartile range. Differences between days were tested using Student’s t-test and the p values are indicated in the figure for each comparison. (B) Correlation between the fraction of neutrophil subtypes and the circulation component of sequential organ failure assessment (SOFA) score. (C) Correlation between the fraction of neutrophil subtypes and SOFA score. (D) Basis component obtained by non-negative matrix factorization (NMF) for Neu1 subtype. Each metagene module is represented by some marker genes. Modules annotated by numbers 1, 2, 3, and 4 corresponds to module A, B, C, and D, respectively. (E–G) Correlation between module B usage and SOFA score. Correlation between module C usage and (F) SOFA and (G) SOFA_circulation_. (H) evaluation of the diagnostic performance of age, Neu1 percentage, and Neu1 modules using receiver operating characteristics curves.

To further explore the clinical relevance of neutrophil subtypes, the correlation between Neu1 fraction and clinical scores was explored. Interestingly, the Neu1 fraction decreased from day 1 to 5, whereas other neutrophil subtypes were increasing or remained constant (Figure 5A). Neu1 fraction was positively correlated with disease severity, which could be explained by the correlation between Neu1 fraction and shock severity (Figures 5B and 5C). The circulation component of the SOFA score declined from day 1–5 (Figure S11). Nonnegative matrix factorization (NMF) was used to identify distinct transcriptional patterns by reducing the high dimension of expression data from hundreds of genes to several metagenes (Brunet et al., 2004). Neu1 gene expression was well captured by four gene expression modules (Figures S12 and S13). Module B was characterized by increased expression of S100A12 and reduced G0S2 and CXCL8 (Figure 5D). Module B usage was positively correlated with SOFA score, and module C usage was negatively correlated with SOFA and SOFA_circulation_ (Figures 5E–5G). The percentage of Neu1 had a moderate diagnostic value for distinguishing shock versus non-shock status (AUC = 0.71), and Neu1_C showed the highest diagnostic performance (AUC = 0.81; Figure 5H).

**Figure 6:**
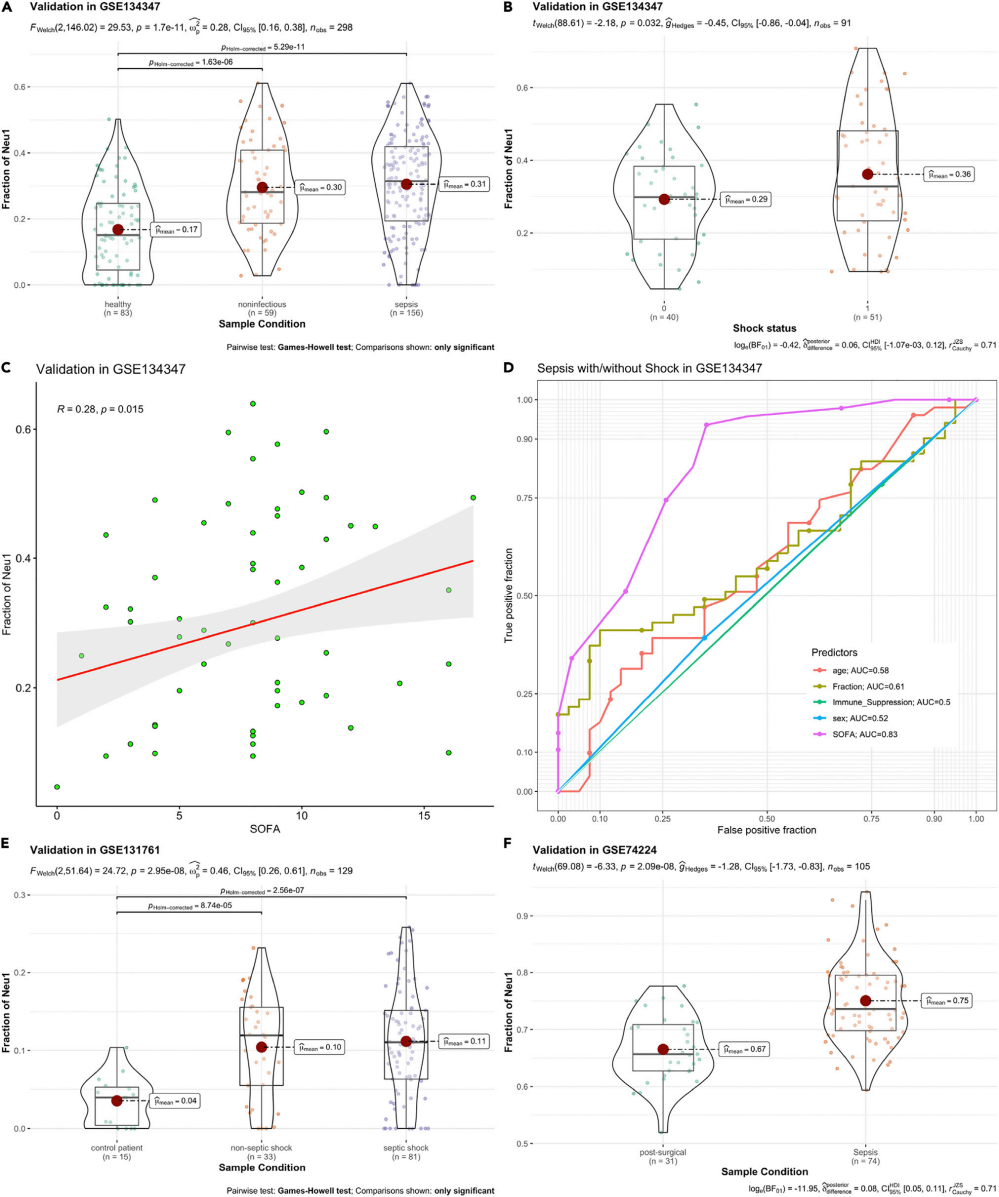
External validation of the role of Neu1 fraction in sepsis severity assessment (A and B) Comparison of Neu1 fraction between different conditions and B) between sepsis with and without shock. Data are represented with violin and box plots indicating the median, and interquartile range. Differences between disease conditions are tested using Welch test and the p values are indicated in the figure for each comparison. (C) Correlation between Neu1 fraction and SOFA in GSE134347 dataset. (D) Diagnostic performance of Neu1 fraction in differentiating sepsis versus septic shock. (E) Comparisons of Neu1 fraction in different clinical conditions. (F) Comparison of Neu1 fraction between surgical versus sepsis conditions. Data are represented with violin and box plots indicating the median and interquartile range. Differences between disease conditions are tested using Welch test and the p values are indicated in the figure for each comparison.

The diagnostic performance of the Neu1 fraction was further validated in external datasets (GSE134347, GSE131761, and GSE74224)(Martínez-Paz et al., 2021; McHugh et al., 2015; Scicluna et al., 2020). These datasets contained bulk transcriptome data from the whole peripheral blood of sepsis patients. The design of these studies varied (Table S3). We deconvoluted the bulk transcriptome data to infer the fraction of each cell type. In the GSE134347 cohort, more Neu1 fraction was observed in sepsis versus healthy controls (Figure 6A), and Neu1 fraction was able to distinguish shock from non-shock sepsis (Figure 6B). There was also a significant correlation between the Neu1 fraction and SOFA score (Figure 6C). The Neu1 fraction had moderate diagnostic performance in diagnosing shock conditions (AUC = 0.61; Figure 6D), which was better than other biomarkers such as age and immune suppression (Figure 6D). Similar results were obtained from datasets GSE131761 and GSE74224 (Figures 6E and 6F). The Neu1 gene expression modules were also tested in the external dataset. The results showed that module C has the highest diagnostic performance in differentiating shock versus non-shock (AUC = 0.80; Figure S14), which was consistent with that in the primary cohort.

### Potential regulatory mechanisms of neutrophil subtypes

**Figure 7:**
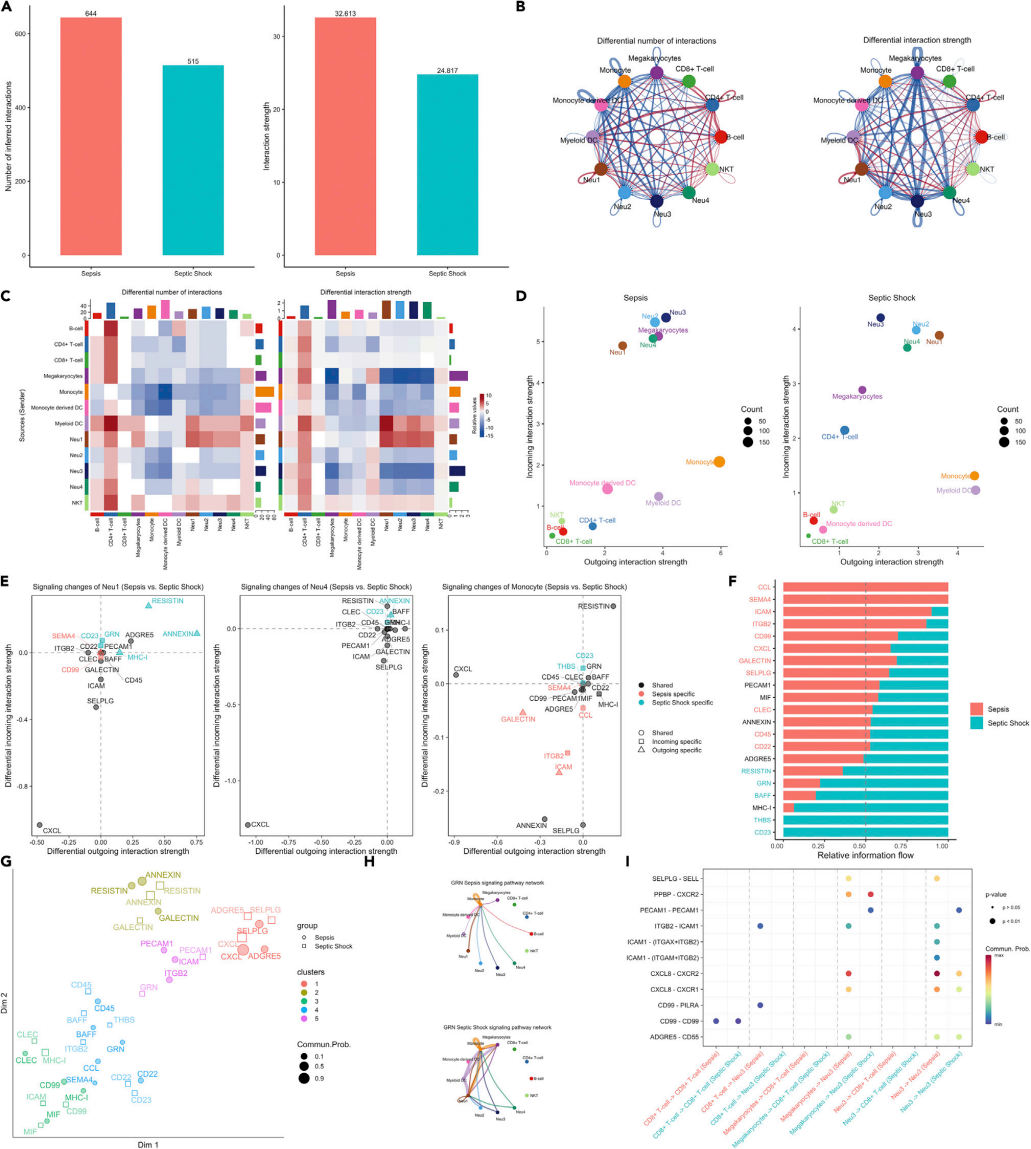
Comparisons of cell-cell communications between sepsis and septic shock (A) The number and strength of inferred communication links between sepsis and septic shock groups. (B) Circle plot showing differential cell-cell communication networks between sepsis and septic shock. The width of edges represents the relative number of interactions or interaction strength. Red (or blue) colored edges represent increased (or decreased) signaling in the septic shock compared to sepsis. (C) Heatmap showing the differential number of interactions or interaction strength in the cell-cell communication network between sepsis and septic shock; red color indicates increased signaling in the septic shock compared to sepsis. The top-colored bar plot represents the sum of the column of values displayed in the heatmap. The right-colored bar plot represents the sum of a row of values. (D) Scatter plots showing the dominant senders (sources) and receivers (targets) in a 2D space. x-axis and y-axis are respectively the total outgoings or incoming communication probability associated with each cell group. Dot size is proportional to the number of inferred links (both outgoing and incoming) associated with each cell group. Dot colors indicate different cell groups. (E) 2D visualization of differential outgoing and incoming signaling associated with one cell group. Positive values indicate the increase in the septic shock dataset whereas negative values indicate the increase in the sepsis dataset. (F) Ranking signaling networks based on the information flow. Significant signaling pathways were ranked based on differences in the overall information flow within the inferred networks between sepsis and septic shock. The top signaling pathways colored red are enriched in sepsis, and these colored green were enriched in septic shock. (G) 2D visualization of the joint manifold learning of signaling networks from sepsis and septic shock datasets. Each dot represents the communication network of one signaling pathway. Dot size is proportional to the overall communication probability. (H) Circle plot showing the inferred GRN signaling network at sepsis and septic shock. Edge width represents the communication probability, and the edge colors are consistent with the color of the sender cell type. (I) Comparisons of significant interactions (Ligand-Receptor pairs) between septic shock and sepsis, which contribute to the signaling from CD4^+^ and CD8^+^T cells to neutrophil subpopulations. Dot color reflects communication probabilities and dot size represents computed p-values. Space means the communication probability is zero. p-values are computed from a one-sided permutation test.

To explore potential regulatory mechanisms of neutrophils, we performed a cell-cell communication analysis. More cell-cell communications in both quantity (644 vs. 515) and strength (44.6 vs. 30.5) were found in non-shock than shock sepsis (Figure 7A). In septic shock, the overall communications between cells involving pro-inflammatory processes were suppressed, consistent with the immunosuppressive profile during severe sepsis (Darcy et al., 2014; Ma et al., 2019; Tai et al., 2018). Communication signals from CD4^+^ and CD8^+^T cells were suppressed comparing septic shock versus sepsis (Figures 7B and 7C). The incoming interaction strength of Neu1 significantly declined in septic shock versus sepsis, whereas the outgoing strength was similar in both conditions (Figure 7D). Specific signaling changes for non-shock versus septic shock included GRN and RESISTIN in Neu1 cells (Figure 7E). These results are consistent with the previous finding that RESISTIN plays an important role in the pathogenesis of sepsis and septic shock (Jang et al., 2017). Next, the information flow for each signaling pathway was compared between sepsis and septic shock. The information flow for a given signaling pathway is defined by the sum of communication probability among all pairs of cell groups in the inferred network. We found that some pathways, including MIF, RESISTIN, GALEXTIN, and PECAM1 maintain a similar flow between conditions (black in Figure 7F). We interpret that these pathways are equally important in sepsis and septic shock. In contrast, other pathways markedly changed their information flow in septic shock as compared to sepsis: (1) turn off (CCL), (2) decrease (such as ICAM, ITGB2), (3) turn on (THBS), or (4) increase (such as APP and BAFF). The signaling pathways associated with inferred networks from both datasets were mapped onto a shared two-dimensional manifold and clustered into groups. Not surprisingly, most of the same pathways from sepsis and septic shock were grouped, including CD22, IL16, MHC-II, and CXCL, indicating these pathways are essential for sepsis response (Figure 7G). However, GRN signaling was classified into different groups, suggesting differential effects of GRN signaling in septic shock versus sepsis (Yan et al., 2016). We specifically examined how GRN communications change between sepsis and septic shock (Figure 7H). In both conditions, monocytes were the dominant source of GRN ligands. Yet, compared to sepsis, when eosinophils and DC equivalently received GRN signals, B-cell gained enhanced responsiveness in septic shock. Ligand GRN and its receptor SORT1 were also found to be active in septic shock, in particular, for the signaling from CD8^+^T cell and Neu1 (Figure 7I). Collectively, the joint manifold learning enables the identification of signaling pathways that undergo severity-dependent changes in sepsis.

### Association of peripheral cell fractions with clinical outcomes in a large critical care database

**Figure 8:**
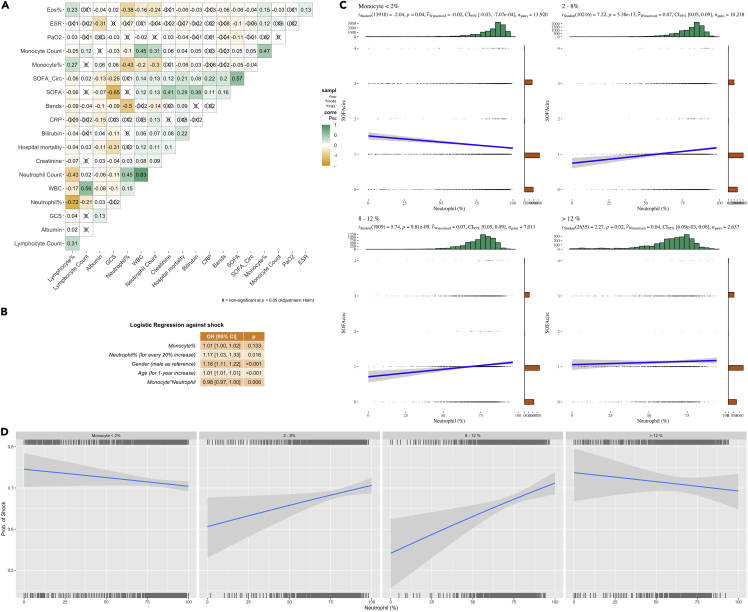
Peripheral blood cell type and clinical outcomes in a large critical care database (A) correlation matrix of cell types and inflammatory biomarkers obtained in routine clinical practice. (B) Multivariable regression model exploring risk factors for septic shock, with an interaction between monocyte and neutrophil fraction. (C) Correlation between neutrophil fraction and SOFA_circulation_ stratified by the monocyte fraction. (D) Association of neutrophil fraction and the probability of shock, stratified by the monocyte fraction. The correlation was adjusted by age and gender.

To validate our findings from scRNA-seq data, we performed big data analytics on a large critical care database comprising > 20,000 sepsis patients(Pollard et al., 2018). SOFA_circulation_ was associated with many peripheral cell fractions in the database such as lymphocytes, monocytes, and bands (person correlation coefficient = 0.2, Figure 8A). Band neutrophils are less mature than segmented neutrophils, which was in line with our finding that the subtype with a less mature score was associated with the clinical severity (i.e., the subtype Neu1 showed a less mature score as compared with other subtypes). Although the overall neutrophil fraction was not associated with the shock severity (SOFA_circulation_), it was associated with the probability of shock and the effects were modifiable by monocyte fraction (Figures 8B and 8C). The negative impact of the neutrophil fraction on septic shock was not observed in the presence of a high or low monocyte fraction, indicating a non-linear interaction between monocyte and neutrophils (Figure 8D).

## Discussion

In this study, we employed scRNA-seq to reveal neutrophil heterogeneity in peripheral blood samples, and its association with clinical severity was examined. Four subtypes of neutrophils were identified in the peripheral blood of sepsis, namely, the Neu1, Neu2, Neu3, and Neu4. Neu1 fractions were found to be an important biomarker for the severity of sepsis, as indicated by the presence of shock. The diagnostic performance was extensively validated in external datasets with bulk RNA transcriptome profiling. First, the proportion of neutrophil subtypes was inferred from the bulk RNA expression profile; and then the association between Neu1 fraction and sepsis severity was explored. The results showed that the Neu1 fraction was significantly associated with the development of sepsis, as well as the disease severity. Furthermore, we identified a gene expression module in the Neu1 subtype that was of moderate diagnostic performance in distinguishing shock versus non-shock conditions. This module was named Neu1_C, comprising signature genes such as NFKBIA, CXCL8, FTH1, and G0S2. However, this module requires further validation before it can be used in clinical practice.

The heterogeneity of neutrophils has been explored in the literature. Grieshaber-Bouyer et al. reported the neutrotime transcriptional signature to define a single continuum of neutrophils across biological compartments, from bone marrow to blood and to target organs (Grieshaber-Bouyer et al., 2021). The results indicated that neutrophil maturation was the key factor responsible for the heterogeneity. Our study also showed that the maturation score differs across the four subtypes of neutrophils. However, neutrophils in the bone marrow and target organs are difficult to obtain in clinical practice, which significantly compromises their clinical utility. Both maturation and aging scores were lower in Neu1 than in other neutrophil subtypes. Further explorations showed that the expansion of premature neutrophils was associated with more severe illness. However, the magnitude of difference in the aging score was small, which can be explained by the fact that all neutrophils were harvested from peripheral blood in our study. It has been reported that the aging scores are significantly different for neutrophils derived from different sites (i.e., bone marrow versus blood)(Xie et al., 2020). Consistent with Xie’s study, neutrophils harvested from peripheral blood can be classified into several subtypes with different apoptosis and aging scores (i.e., The G5a subtype may correspond to the Neu1 in our study) (Xie et al., 2020).

Interleukin-1 signaling pathways have been widely explored for their association with sepsis severity in the literature. However, our study failed to identify significantly enriched pathways involving IL-1. The inconsistency can be explained in the following aspects. First, the sample size in our study is small, which lacks statistical power to detect some important pathways. Second, many of previous studies are measuring plasma protein levels of the IL-1 signaling pathway (Meyer et al., 2018; Shakoory et al., 2016). Our study measured RNA within cells, and intracellular RNA levels may not directly correlate to the plasma protein levels.

The observation that Neu1 was associated with septic shock was also supported by several other findings. First, a mature score of Neu1 was found to be smaller than other neutrophil types and may contain more bands than other subtypes; Neutrophil bands were found to be positively correlated with the shock severity in the eICU-CRD cohort involving more than 20,000 sepsis patients. Second, Neu1 expressed a high level of OLFM4 compared to other neutrophil subtypes (Figure S5). Using microarray data, Martínez-Paz P et al. found that OLFM4 was upregulated in septic shock versus non-shock(Martínez-Paz et al., 2021). Furthermore, several studies showed that the abundance of neutrophil subtypes with high expression of OLFM4 was associated with the severity of sepsis (Kangelaris et al., 2021; Kassam et al., 2021). Third, the strength of the association between Neu1 and clinical severity was modifiable by the presence of other cell types such as monocyte. The negative impact of the neutrophil fraction on disease severity was not observed in the presence of a high monocyte fraction, suggesting the importance of the interaction between monocyte and neutrophils in the pathogenesis of sepsis (Udovicic et al., 2021).

Neu1 expansion was associated with an increased risk of septic shock. The biological functions of this subtype were extensively studied in the study. Compared with the results in the literature, the Neu1 subtype corresponds to the less mature neutrophils. Meghraoui-Kheddar et al. reported two subtypes of neutrophils in the peripheral blood of sepsis and found that the less mature subtype correlated well with clinical severity (Meghraoui-Kheddar et al., 2022). CD64 (FCGR1A) expression in neutrophils has been widely investigated for its diagnostic performance in distinguishing sepsis versus non-sepsis (Elawady et al., 2014, p. 64; Hoffmann, 2009, p. 64). However, our study did not show evidence that CD64 was more highly expressed in the Neu1 subtype, suggesting its limited role in distinguishing shock versus non-shock conditions. Some cell surface biomarkers were predicted by using machine learning approaches. Specifically, we found neutrophil subtypes with high expressions of CD123, CD38, and CD69 were associated with a worse prognosis. This finding is consistent with evidence from other studies (Goswami et al., 2021; Zhou et al., 2019). Meghraoui-Kheddar et al. identified a subset of neutrophils with high CD123 expression, which is also associated with disease severity (Meghraoui-Kheddar et al., 2022). The surface biomarker CD38 was more likely to be expressed in common myeloid progenitors, whose expansion in peripheral blood may indicate severe sepsis (Wang et al., 2020).

### Limitations of the study

However, our study lacks a translational approach/therapeutic intervention in experimental design. Some potential clinical implications of the study are the identification of the neutrophil subtype with prognostic implications, and therapeutic intervention targeting the molecular pathways involving Neu1. These require further experimental trials to validate.

In summary, our study establishes a general framework for studying neutrophil-related mechanisms, prognostic biomarkers, and potential therapeutic targets for septic shock.

## STAR★Methods

### Key resources table

**Table:** 

REAGENT or RESOURCE	SOURCE	IDENTIFIER
**Deposited data**		

Single-cell RNA-seq data	https://ngdc.cncb.ac.cn	PRJCA006118
Sepsis Bulk RNA-seq	https://www.ncbi.nlm.nih.gov/geo/	GSE134347
Sepsis Bulk RNA-seq	https://www.ncbi.nlm.nih.gov/geo/	GSE131761
Sepsis Bulk RNA-seq	https://www.ncbi.nlm.nih.gov/geo/	GSE74224

**Software and algorithms**		

R (version 4.1.1)	https://cran.r-project.org/	R CRAN
CellChat	http://www.cellchat.org/	CellChat
cTP-net	https://github.com/zhouzilu/cTPnet	cTP-net

### Resource availability

#### Lead contact

Further information and requests for resources and reagents should be directed to and will be fulfilled by the lead contact, Zhongheng Zhang (zh_zhang1984@zju.edu.cn).

#### Materials availability

This study did not generate new unique reagents.

### Experimental model and subject details

The study cohort comprised subjects with a confirmed diagnosis of sepsis who presented to the emergency department (ED) of four tertiary care hospitals. Sepsis was defined as suspected or documented infection plus an acute increase in SOFA score >2 points (Singer et al., 2016). Sepsis patients were excluded if they met one of the following criteria: 1) concomitant malignancy, autoimmune disease; 2) pregnancy; 3) end-stage cirrhosis with Child-Pugh C; 4) patients who signed do-not-resuscitate order; 5) sepsis onset >48 h when presenting to the participating hospitals; 6) immunosuppression such as long-term use of corticosteroids, immunosuppressive agents, chemotherapy, radiotherapy or HIV infection; and 7) acute myocardial infarction and/or pulmonary embolism. Septic shock was defined as those who required vasopressors to maintain arterial mean blood pressure over 60 mmHg despite adequate fluid resuscitation. Clinical data and blood samples were collected on days 1, 3, and 5 after ED admission. Blood samples were drawn into EDTA tubes, stored, and transported at 4–8°C as soon as possible. The nucleated cells of blood samples were separated within 6 h. The study was approved by the ethics committee of Sir Run Run Shaw hospital. Written informed consent was obtained from all patients or their surrogates.

The sex and gender do not significantly influence the results as we have adjusted for these covariates. The age range of included subjects was 52 to 90 years, with 9 males and 3 females.

### Method details

#### Singleron Matrix^TM^ single-cell RNA sequencing

Cells were isolated from whole-blood samples, and 2 mL GEXSCOPE™ red blood cell lysis buffer (Singleron) was added at 25°C for 10 min. Single-cell suspensions with 1×10^5^ cells/mL in concentration in PBS (HyClone) were prepared. Single-cell suspensions were then loaded onto microfluidic devices and scRNA-seq libraries were constructed according to Singleron GEXSCOPE® protocol by GEXSCOPE® Single-Cell RNA Library Kit (Singleron Biotechnologies). Individual libraries were diluted to 4nM and pooled for sequencing. Pools were sequenced on Illumina HiSeq X with 150 bp paired end reads.

#### scRNA-seq quantifications and statistical analysis

Raw reads were processed to generate gene expression profiles using an internal pipeline. Briefly, after filtering reads without poly T tails, cell barcode and UMI was extracted. Adapters and poly A tails were trimmed (fastp V1) before aligning read two to GRCh38 with ensemble version 92 gene annotation (fastp v2.5.3a and featureCounts v1.6.2)(Liao et al., 2014). Reads with the same cell barcode, UMI and gene were grouped together to calculate the number of UMIs per gene per cell. The UMI count tables of each cellular barcode were used for further analysis. Celltype identification and clustering analysis using Seurat (v4.0.4) and clustermole (v1.1.0) (Butler et al., 2018; Satija et al., 2015). Rigorous quality control was performed and poor-quality cells with the number of gene features >2500 or <200, percent of mitochondria genes >10% were removed. Standard Seurat workflow analysis was performed. Different samples were integrated with the IntegrateData function (Butler et al., 2018). The parameter resolution was set to 0.15 for FindClusters function to identify clusters (Figure S2). Differentially expressed genes (DEGs) between different clusters were identified with function FindMarkers, and gene set enrichment analysis (GSEA) was performed to identify enriched pathways (Subramanian et al., 2005).

Subtypes of neutrophils were identified by a second-step clustering with a resolution of 0.2. The resolution was determined by the adjusted Rand index as described in the literature (Reyes et al., 2020). Briefly, 90% of total neutrophil cells were randomly sampled without replacement for 20 iterations. Clustering was repeated in each subsample, and an adjusted Rand index was then computed between the solutions for the original and subsampled data. The highest resolution at which the Rand index began to decline was used for downstream analysis. The cell surface protein abundance is imputed from single-cell transcriptomes by deep neural networks, by the tool single cell Transcriptome to Protein prediction with deep neural network (cTP-net) (Zhou et al., 2020).

#### Biological functions of neutrophil subtypes

Individual neutrophil cells were scored for their expression of signature genes involving certain biological functions as described elsewhere (Xie et al., 2020). The normalized expression of signature genes was averaged to obtain each functional score. The signature expressions of all relevant biological functions were displayed in a heatmap. A weighted average of Z scores (scaling the normalized expression of a gene across all cells) of age-related genes was calculated to score the aging condition. Either 1 or −1 was set to the weight to indicate positive or negative relationships. Granule signature genes were obtained from the reference (Cowland and Borregaard, 2016). The neutrophil maturation signature genes were derived by as described in the literature (Xie et al., 2020). Other functional signatures were derived from the Gene Ontology database (The Gene Ontology Consortium, 2017). The integrated proapoptotic pathway was used to measure the apoptotic score. A two-component Gaussian mixture model was exploited to the apoptotic score of all neutrophil cells using the R package *mclust* (version 5.4.7), so that apoptotic heterogeneity in peripheral neutrophil populations was dissected independently of transcriptome-based clustering (Figure S6). The component with higher mean score was designated as the apoptotic group and each cell was assigned to one of the two groups based on its posterior distribution.

#### Deconvolution of bulk transcriptome data and external validation

The fractions of neutrophil subtypes were compared between patients with and without shock, and the association between neutrophil subtype fraction and circulation component of SOFA score (SOFA_circulation_) was tested using the Persons’ correlation test. Discrimination of neutrophil subtype for shock versus non-shock was explored with the area under the receiver operating characteristic curve (AUC). The gene expression module of type 1 neutrophil (Neu1) was explored using the non-negative matrix factorization. The best number of ranks was determined collectively by statistics including cophenetic coefficient, RSS, dispersion, and silhouette (Berry et al., 2007). The correlation between the Neu1 module and SOFA score was explored to dissect metagenes associated with the disease severity.

We retrieved external bulk transcriptome profiling for sepsis patients from the GEO database (GSE134347(Scicluna et al., 2020), GSE131761(Martínez-Paz et al., 2021), and GSE74224 (McHugh et al., 2015)) to validate the association of neutrophil subtype fraction and sepsis severity. The bulk transcriptome data were deconvoluted by using the CIBERSORT algorithm (Newman et al., 2015), so that the fraction of each cell component can be estimated for the bulk transcriptome samples. The clinical phenotypes including clinical conditions (sepsis vs. surgical), SOFA, and shock were extracted if available. The correlation between Neu1 and severity score and the discrimination power of the Neu1 fraction were explored to validate the results from our primary cohort.

#### Cell-cell communication inference and intercellular communication networks

Cell-cell communication inference was performed using the CellChat (http://www.cellchat.org/) workflow (Jin et al., 2021). The probability of cell-cell communications was inferred by integrating the gene expression profile of each cell and prior knowledge of interactions between receptors, ligands, and their cofactors. The label-based mode was employed with cells labeled by the cell types obtained from the above Seurat workflow. Neutrophil subtypes were used in the cell label scheme. The curated signaling molecule interaction database CellChatDB was used to assign roles for the signaling molecules and their interactions. Significant cell communications were obtained by identifying differentially over-expressed ligands and receptors for each cell group. The global communication patterns and key signals in different cell groups were identified by a pattern recognition method based on non-negative matrix factorization.

#### Correlation between peripheral cell fraction with clinical phenotypes in a large clinical dataset

The eICU Collaborative Research Database (eICU-CRD) was screened for potentially eligible patients who met the diagnostic criteria of sepsis (Pollard et al., 2018). The peripheral blood cell counts and fractions were extracted from the laboratory events table from day 1 to day 10 after ICU admission. The shock status was defined as SOFA_circulation_> 1 point. Persons’ correlation between prespecified biomarkers was explored in a correlation matrix. A multivariable logistic regression model was established to investigate the modification effect of monocyte or eosinophil on neutrophil fraction, adjusting for sex and age (Zhang, 2016). The modification effects were visualized with the *visreg* (v2.7.0) function (Breheny and Burchett, 2017). The correlations between SOFA_circulation_ and neutrophil, stratified by monocyte or eosinophil were also explored by conventional methods.

### Quantification and statistical analysis

The differences in cell fractions between days are tested using the Student t test. Quantitative data were represented with box plot which showed median, interquartile range, and outliers. The neutrophil subtype fractions were represented with box plot indicating the median, and interquartile range. Differences in neutrophil subtypes between days were tested using the Student t test. In external validation of Neu1 fraction, the Neu1 fraction was represented with violin and box plots indicating the median, and interquartile range. Differences between disease conditions are tested using the Welch test and the p values were corrected for multiple comparisons using the Bonferroni-Holm method. All analyses were performed using R (version: 4.1.1).

### Additional resources

None.

## Data Availability

• Single-cell RNA-seq data have been deposited at the National Genomic Data Center and GEO repository (see key resources table). • This paper does not report the original code. • Any additional information required to reanalyze the data reported in this paper is available from the lead contact upon request.
