# Supplementary material for: Single-cell transcriptome profiling reveals heterogeneous neutrophils with prognostic values in sepsis
Source: iScience. 2022 Oct 7;25(11):105301. doi: 10.1016/j.isci.2022.105301 (PMC9593767; doi:10.1016/j.isci.2022.105301)
Supplement: Document S1. Figure S1–S14 and Tables S1–S3 [file mmc1.pdf]

iScience, Volume *25*

Supplemental information

Single-cell transcriptome profiling reveals heterogeneous neutrophils with prognostic values in sepsis

Yucai Hong, Lin Chen, Jian Sun, Lifeng Xing, Yi Yang, Xiaohong Jin, Huabo Cai, Lianlian Dong, Liping Zhou, and Zhongheng Zhang

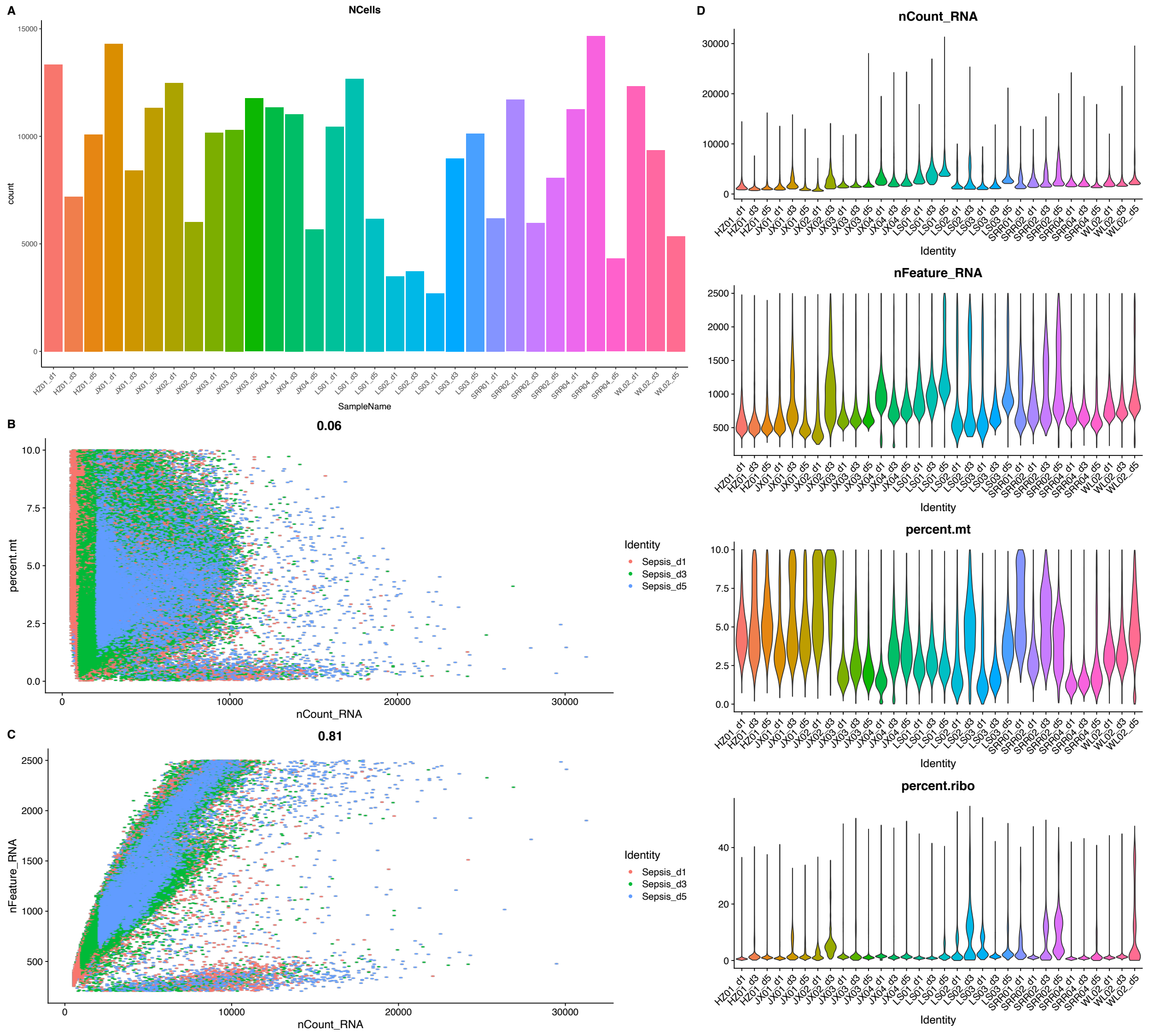

Figure S1. Quality control of included cells, Related to STAR Methods

A) bar plot showing the number of cells for each sample. B) scatter plots showing the relationship between the percentage of mitochondria genes B) or the number of unique gene features C) and the number of RNA count; the points are colored by the disease conditions as sepsis days 1, 3 and 5. D) violin plots showing the number of RNA counts, the number of unique RNA features, percent of mitochondria genes, and ribosome genes.

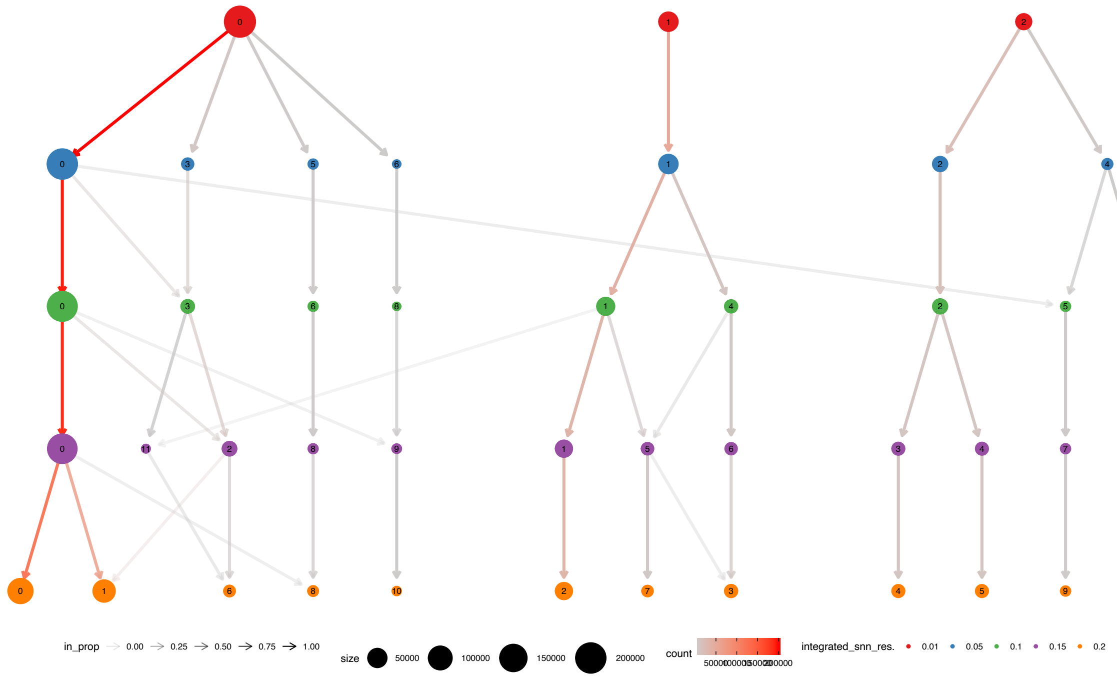

Figure S2. Cluster tree showing the classification of the cell population with increasing resolution from 0.01 to 0.2. Related to STAR Methods

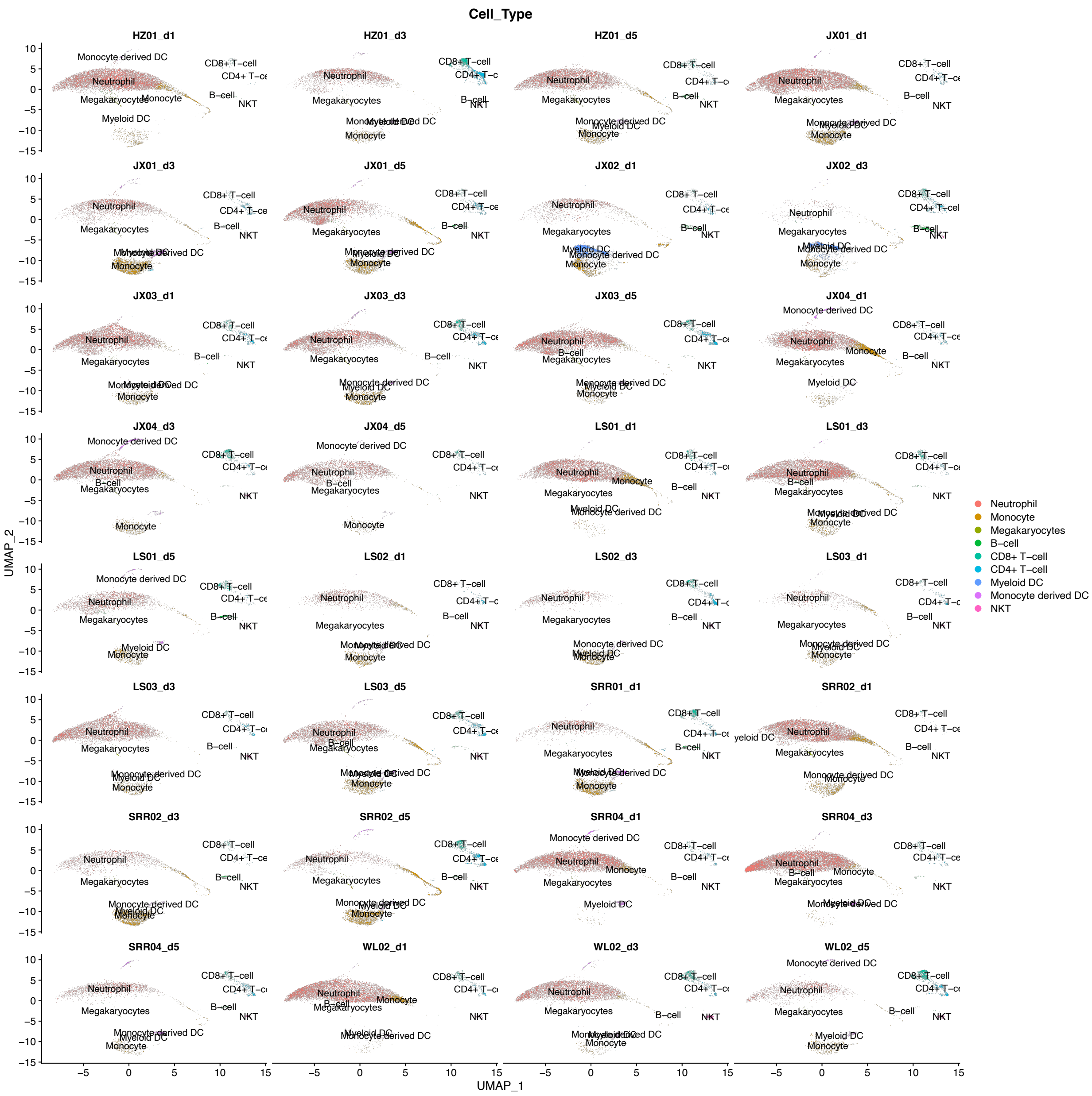

Figure S3. Projection of blood cells onto the 2-dimensional UMAP space stratified by sample identity. Related to Figure 2.

Table S1 Marker genes for neutrophil cluster as compared to all other cell types, Related to Figure 2

Gene	p_val	avg_log2FC	pct.1	pct.2	p_val_adj	cluster
FCGR3B	2.180560e-205	1.9623092	0.992	0.921	4.361120e-202	Neutrophil
SRGN	1.596438e-141	0.9275004	0.999	0.988	3.192875e-138	Neutrophil
SLC25A37	1.918087e-127	1.5203271	0.970	0.963	3.836175e-124	Neutrophil
CXCR2	2.696403e-120	1.8465643	0.938	0.873	5.392807e-117	Neutrophil
CD177	9.192438e-119	1.6019222	0.998	0.996	1.838488e-115	Neutrophil
ALPL	1.256831e-102	1.7914833	0.911	0.909	2.513661e-99	Neutrophil
G0S2	3.712930e-96	1.2319299	0.925	0.744	7.425860e-93	Neutrophil
IL1R2	1.798265e-90	1.5770026	0.998	0.995	3.596530e-87	Neutrophil
CEACAM1	1.846631e-79	0.5401477	0.970	0.981	3.693261e-76	Neutrophil
S100A9	3.465762e-79	0.6603747	1.000	1.000	6.931524e-76	Neutrophil
S100A12	3.286343e-71	0.6157729	1.000	1.000	6.572686e-68	Neutrophil
ANXA3	1.732947e-65	1.1849449	0.991	0.994	3.465895e-62	Neutrophil
CST7	4.527322e-64	1.0586913	0.926	0.938	9.054644e-61	Neutrophil
S100A8	4.792402e-64	0.5837515	1.000	1.000	9.584803e-61	Neutrophil
CMTM2	3.213146e-60	1.3180151	0.804	0.720	6.426293e-57	Neutrophil
MMP9	3.098238e-50	1.1105493	0.972	0.981	6.196476e-47	Neutrophil
ZDHHC19	1.688474e-44	1.2023346	0.856	0.885	3.376948e-41	Neutrophil
NFKBIA	5.781407e-35	0.7303813	0.959	0.923	1.156281e-31	Neutrophil
FCER1G	2.664707e-34	0.7597350	0.998	0.986	5.329414e-31	Neutrophil
IFITM3	1.199335e-33	0.7348638	0.972	0.919	2.398671e-30	Neutrophil
AC023157.3	2.210213e-33	0.3953758	0.277	0.449	4.420426e-30	Neutrophil
HPGD	2.833890e-30	0.4208696	0.684	0.608	5.667779e-27	Neutrophil
FTH1	9.155820e-27	0.3800970	0.996	0.983	1.831164e-23	Neutrophil
APOBEC3A	3.788842e-25	0.7391981	0.899	0.813	7.577684e-22	Neutrophil
SOCS3	5.147121e-24	0.5282766	0.846	0.648	1.029424e-20	Neutrophil
AC084871.2	1.319175e-20	0.4146843	0.273	0.390	2.638350e-17	Neutrophil
S100P	2.713500e-20	0.9290111	0.905	0.960	5.426999e-17	Neutrophil
SLPI	6.158300e-13	0.9184458	0.461	0.636	1.231660e-09	Neutrophil
CXCL8	4.659280e-11	0.5304183	0.764	0.696	9.318560e-08	Neutrophil
SYNE2	9.920946e-10	0.4320792	0.410	0.556	1.984189e-06	Neutrophil
TNFAIP6	1.074663e-09	0.7108396	0.957	0.973	2.149325e-06	Neutrophil
RGL4	2.529223e-09	0.7315480	0.935	0.977	5.058445e-06	Neutrophil
IER3	3.534100e-09	0.8340195	0.532	0.419	7.068200e-06	Neutrophil
ADM	4.248100e-08	0.8605398	0.769	0.757	8.496200e-05	Neutrophil
IL1B	2.537993e-07	0.4437119	0.655	0.602	5.075986e-04	Neutrophil
GADD45A	5.949040e-07	0.5655661	0.791	0.874	1.189808e-03	Neutrophil
CXCL1	7.182611e-07	0.5807764	0.473	0.663	1.436522e-03	Neutrophil
ORM1	6.655010e-05	0.3369707	0.803	0.869	1.331002e-01	Neutrophil
ANKRD22	7.152097e-05	0.2864393	0.908	0.947	1.430419e-01	Neutrophil
HIST1H2AC	9.250242e-05	0.6596016	0.655	0.830	1.850048e-01	Neutrophil
NFE4	4.444879e-04	0.5862805	0.474	0.628	8.889758e-01	Neutrophil
TNFAIP3	4.911991e-04	0.6508646	0.716	0.768	9.823982e-01	Neutrophil
GADD45B	7.676633e-04	0.4515092	0.671	0.592	1.000000e+00	Neutrophil
IL1RN	1.207308e-03	0.9676301	0.842	0.931	1.000000e+00	Neutrophil
RBP7	1.207985e-03	0.2948473	0.433	0.426	1.000000e+00	Neutrophil

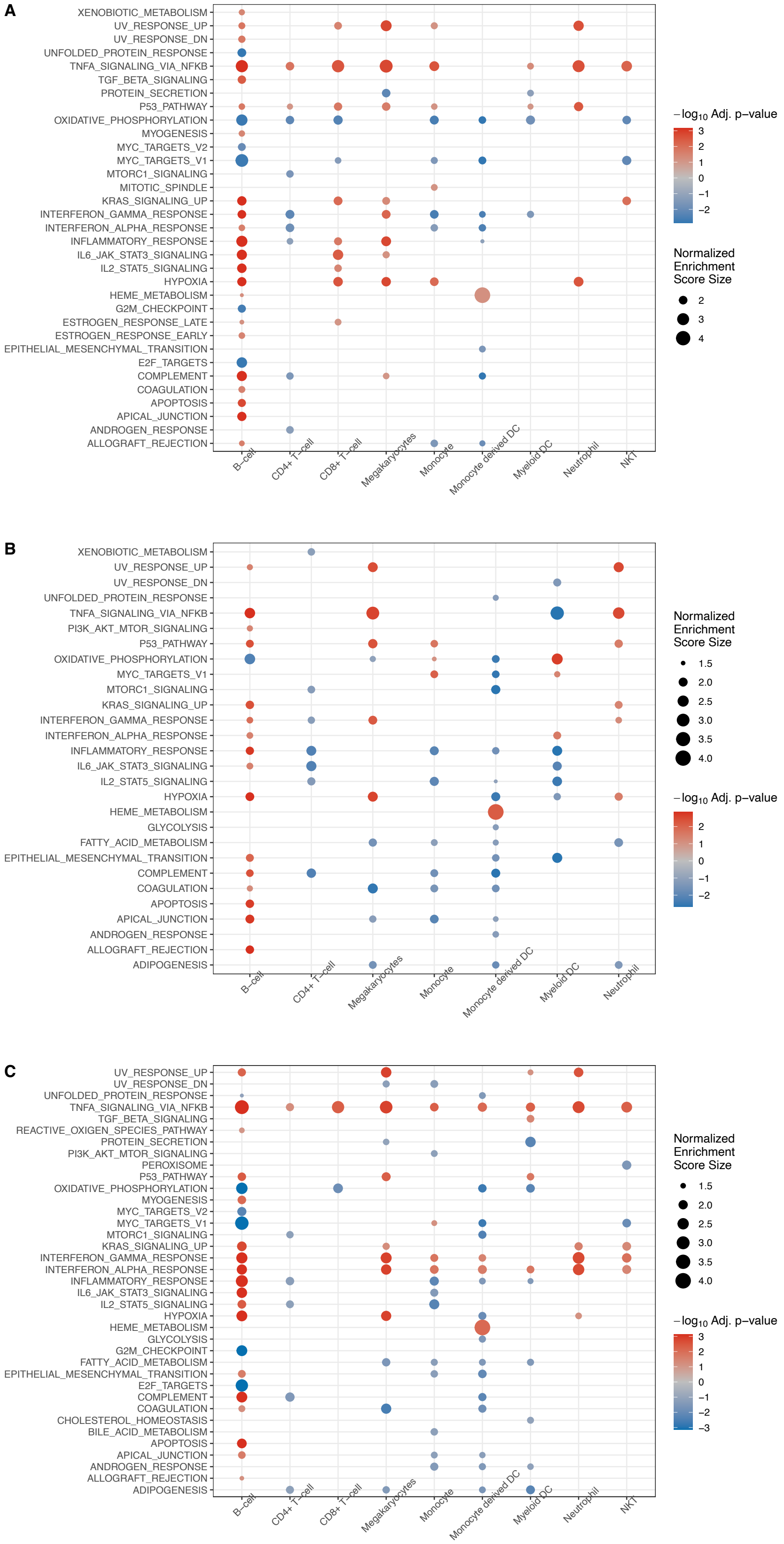

Figure S4. GO enrichment analysis for marker genes in each cell type between mild versus severe sepsis stratified by disease course on day 1 (A), 3 (B) and 5 (C). Related to Figure 2. The negative value indicates downregulated pathways.

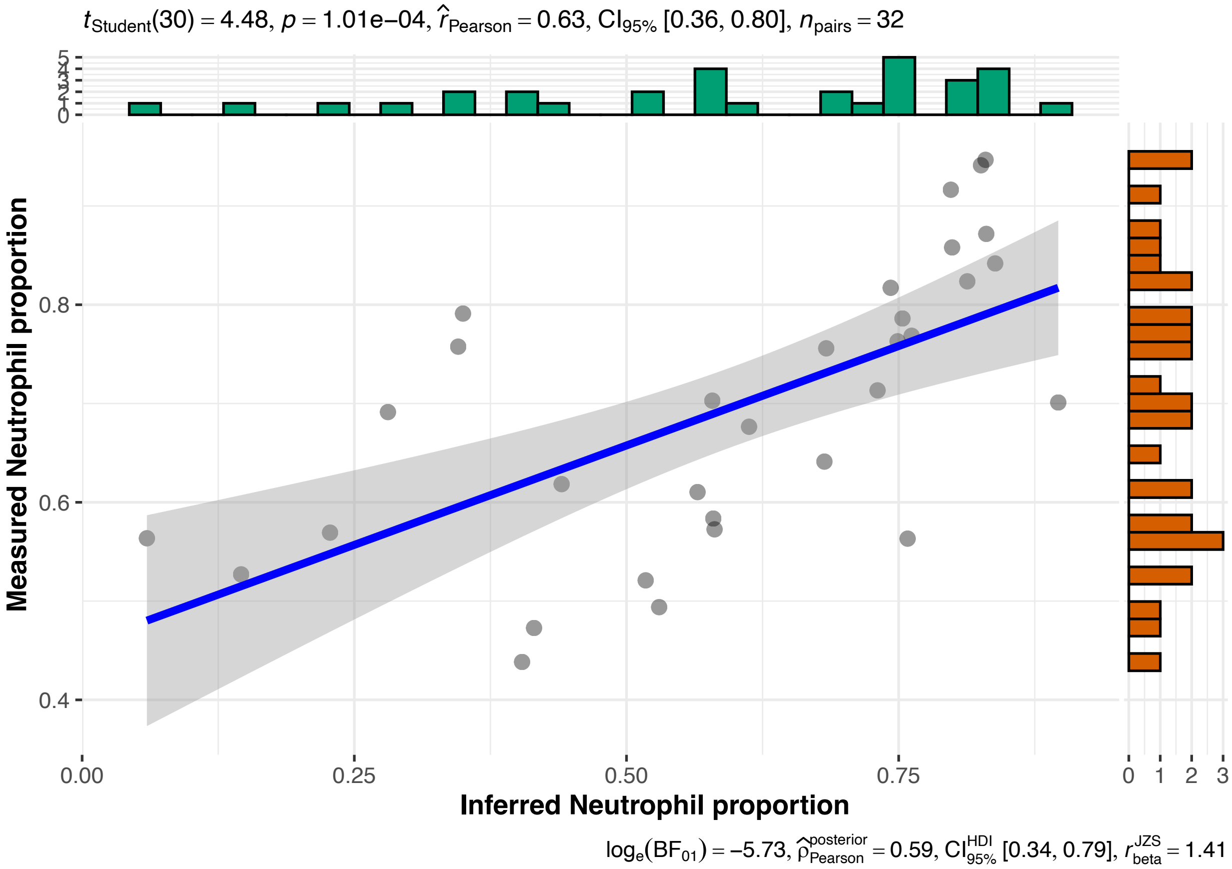

Figure S5 Correlation between inferred neutrophil proportion and measured neutrophil proportion. Related to Figure 2.

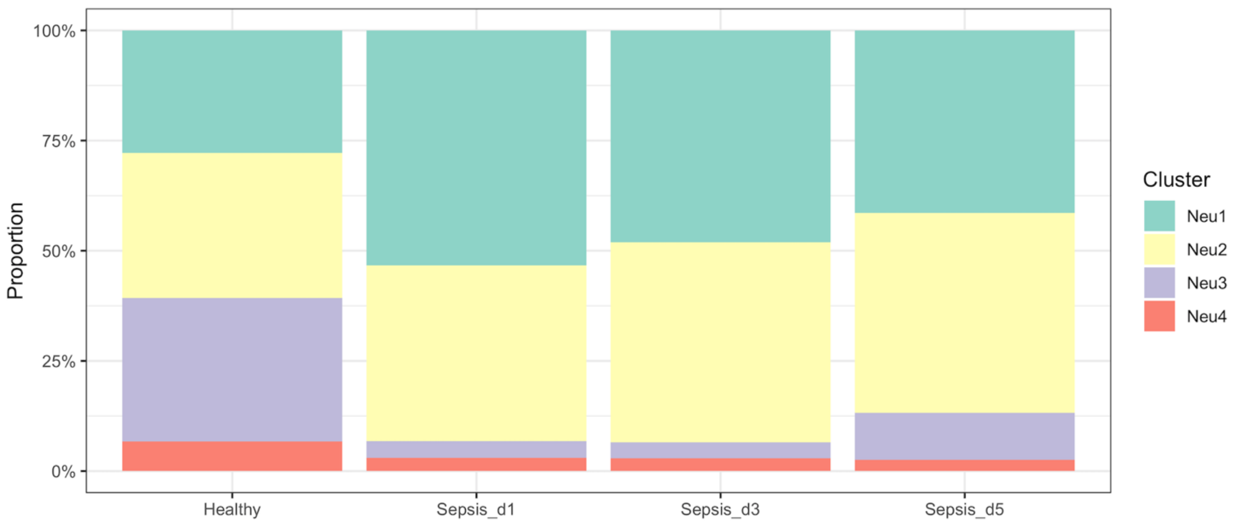

Figure S6. Fraction of neutrophil subtypes across different sepsis days as well as in the healthy control subject. Related to Figure 3.

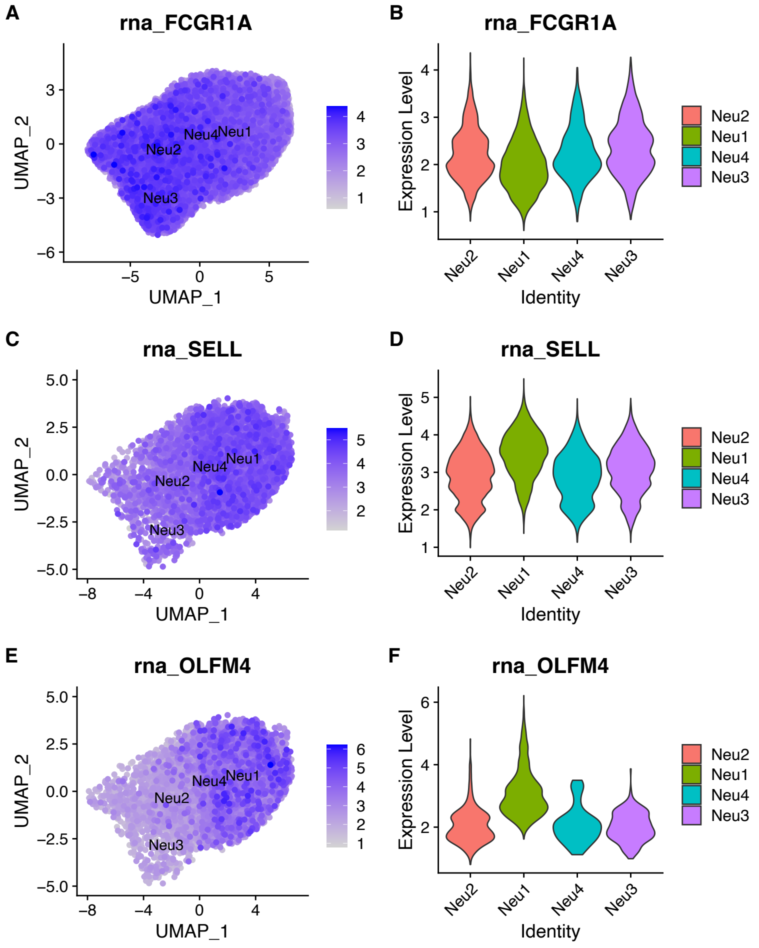

Figure S7. The expression of some genes across subtypes of neutrophils. Related to Figure 3.

The results indicated that FCGR1A (CD64) was not significantly different across neutrophil subtypes (A and B); and SELL (CD62L) was more highly expressed in Neu1 than other types (C and D); OLFM4 was more highly expressed in Neu1, as compared to other subtypes (E and F).

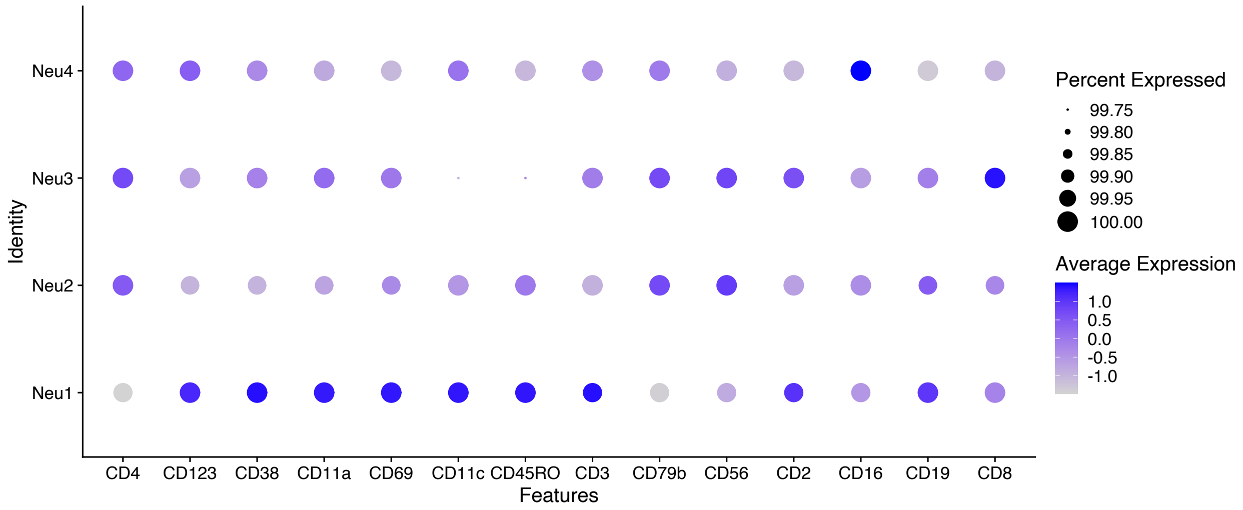

Figure S8. Cell surface biomarker for neutrophil subtypes. Related to Figure 3.

The cell surface protein abundance is imputed from single-cell transcriptomes by deep neural networks, by the tool single cell Transcriptome to Protein prediction with deep neural network (cTP-net). Then the protein abundance was compared between different subtypes of neutrophils using Wilcoxon Rank Sum test.

.

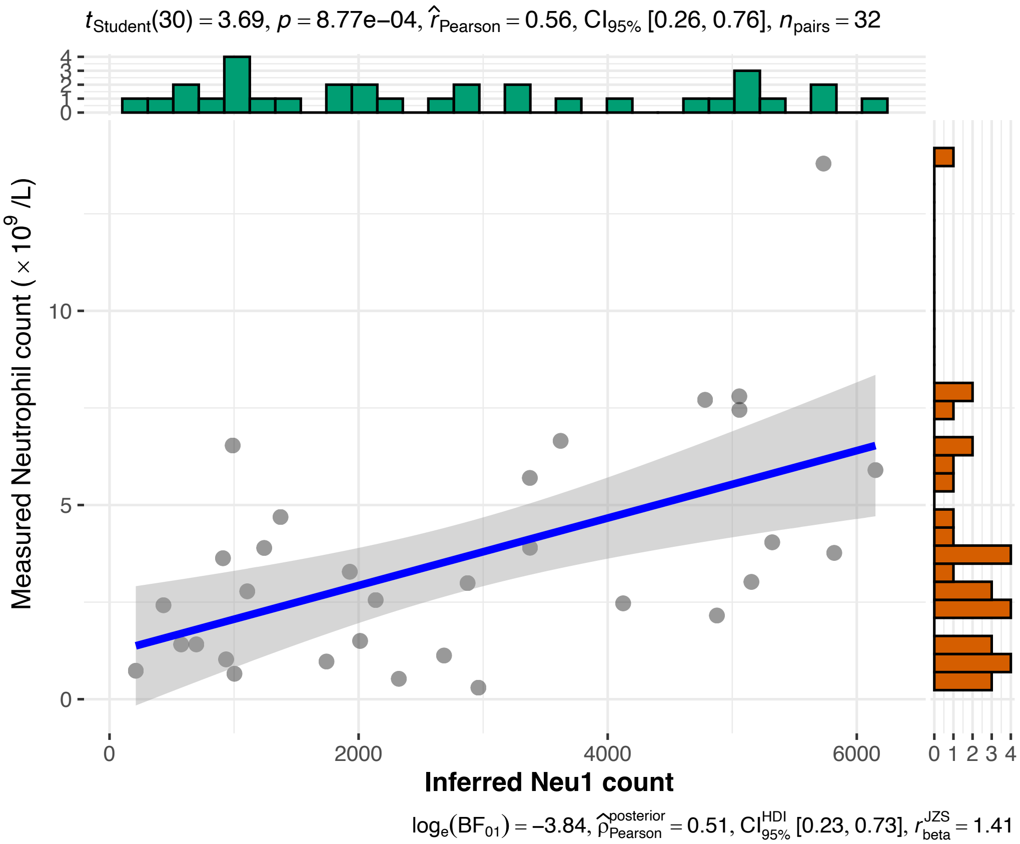

$t_{Student}(30) = 3.69$, $p = 8.77e{-}04$, $\hat{r}_{Pearson} = 0.56$, $CI_{95\%}$ [0.26, 0.76], $n_{pairs} = 32$
Measured Neutrophil count ($\times 10^9$/L)
Inferred Neu1 count
0
2000
4000
6000
0
5
10
$\log_e(BF_{01}) = -3.84$, $\hat{\rho}^{posterior}_{Pearson} = 0.51$, $CI^{HDI}_{95\%}$ [0.23, 0.73], $r^{JZS}_{beta} = 1.41$

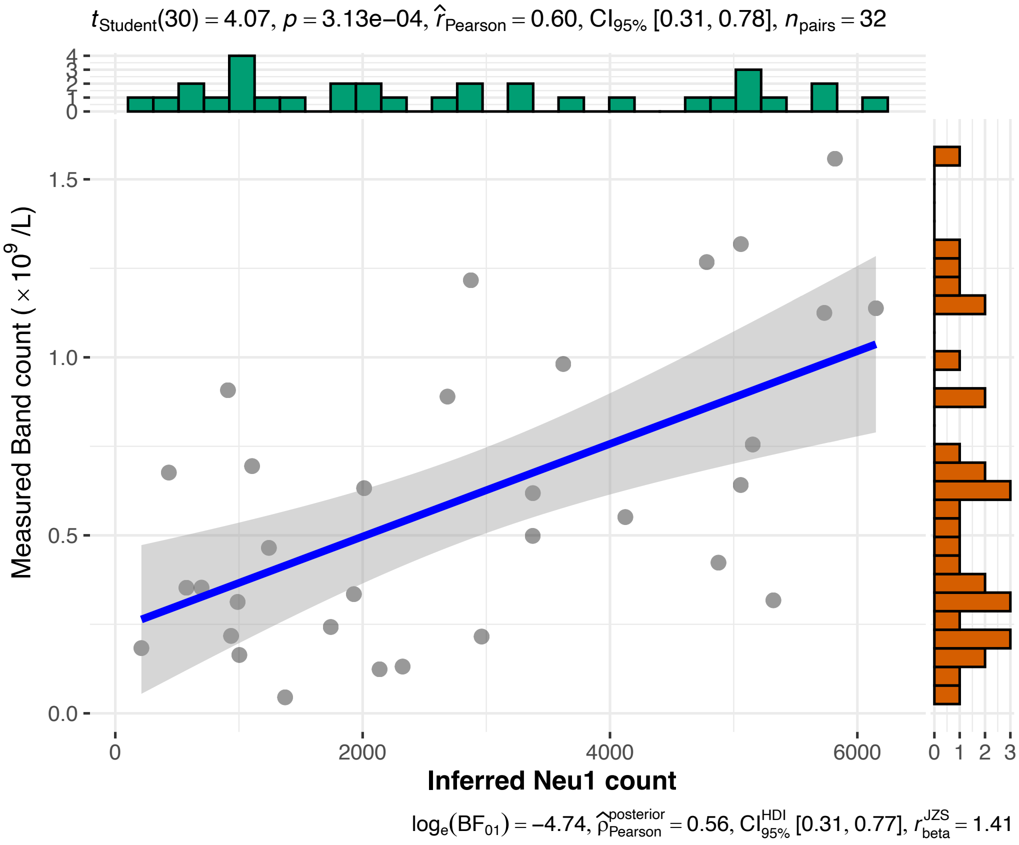

$t_{Student}(30) = 4.07$, $p = 3.13e{-}04$, $\hat{r}_{Pearson} = 0.60$, $CI_{95\%}$ [0.31, 0.78], $n_{pairs} = 32$
Measured Band count ($\times 10^9$/L)
Inferred Neu1 count
0
2000
4000
6000
0.0
0.5
1.0
1.5
$\log_e(BF_{01}) = -4.74$, $\hat{\rho}^{posterior}_{Pearson} = 0.56$, $CI^{HDI}_{95\%}$ [0.31, 0.77], $r^{JZS}_{beta} = 1.41$

Figure S9 Correlation between inferred Neu1 count and measured (A) neutrophil and (B) band counts. Related to Figure 3.

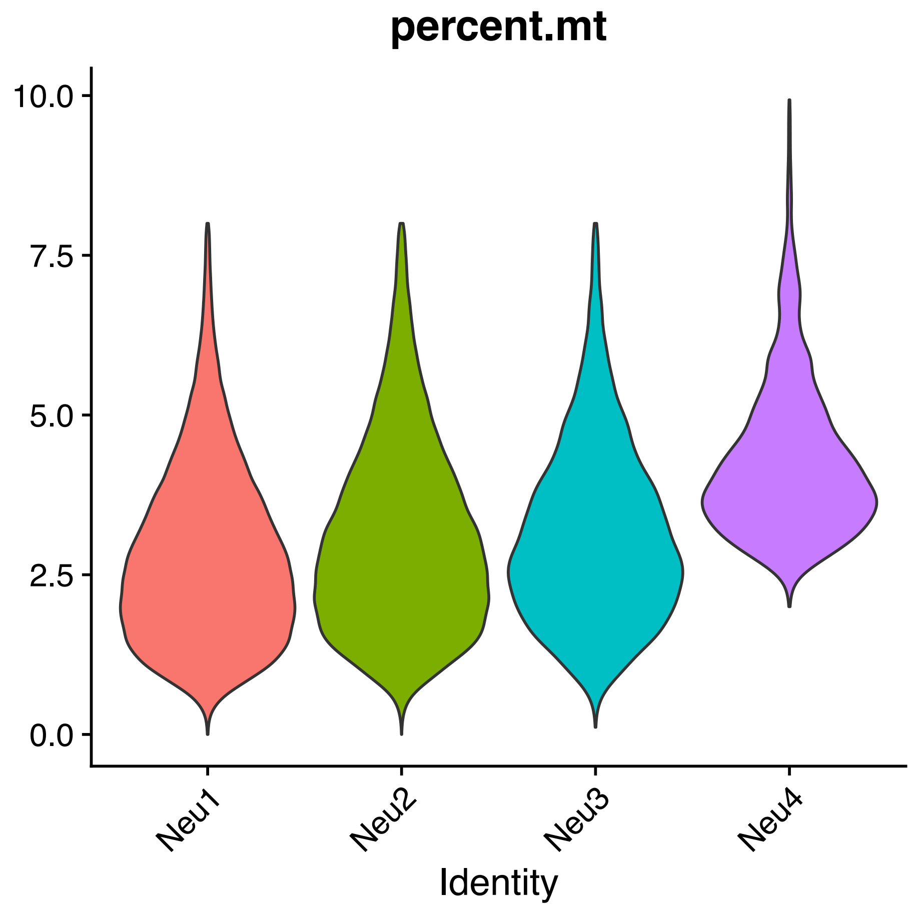

Figure S10. Comparisons of the percentage of mitochondrial gene in subtypes of neutrophils. Related to Figure 4.

Table S2 Marker genes for neutrophil subtype Neu1 as compared against all other neutrophil subtypes, Related to Figure 4.

gene	p_val	avg_log2FC	pct.1	pct.2	p_val_adj	cluster
MMP9	3.413340e-240	1.8713925	0.820	0.370	8.365755e-236	Neu1
S100A8	1.788253e-204	0.7109580	1.000	1.000	4.382829e-200	Neu1
S100A12	1.199535e-187	1.2561568	0.993	0.932	2.939941e-183	Neu1
S100A9	2.558593e-122	0.5268207	1.000	1.000	6.270855e-118	Neu1
CD177	3.919329e-93	0.8122266	0.854	0.630	9.605882e-89	Neu1
GAPDH	1.004298e-65	0.6017680	0.864	0.662	2.461435e-61	Neu1
ALOX5AP	3.843562e-63	0.6044534	0.925	0.775	9.420187e-59	Neu1
VIM	3.463708e-57	0.5891169	0.922	0.746	8.489201e-53	Neu1
CST7	1.309729e-40	0.4009172	0.887	0.713	3.210014e-36	Neu1
TSPO	6.070338e-35	0.4129562	0.812	0.663	1.487779e-30	Neu1
GCA	3.430734e-33	0.3577926	0.930	0.848	8.408385e-29	Neu1
S100A6	9.761154e-32	0.3064234	0.981	0.949	2.392361e-27	Neu1
MYL6	3.255652e-30	0.3809760	0.924	0.828	7.979276e-26	Neu1
ACTB	2.032315e-28	0.2157136	0.999	0.997	4.981001e-24	Neu1
SLC2A3	1.800724e-24	0.3717823	0.835	0.691	4.413395e-20	Neu1
SH3BGRL3	1.880507e-16	0.2066257	0.863	0.753	4.608936e-12	Neu1
MT-CO3	1.170802e-15	0.3257162	0.824	0.718	2.869518e-11	Neu1
CAP1	4.403561e-15	0.2659042	0.886	0.789	1.079269e-10	Neu1
S100A4	1.960162e-13	0.2332916	0.916	0.866	4.804162e-09	Neu1
KLF6	3.133953e-12	0.2143451	0.822	0.711	7.681006e-08	Neu1
TPT1	3.720214e-12	0.1503707	0.891	0.825	9.117873e-08	Neu1
CFL1	4.960670e-11	0.1829016	0.816	0.722	1.215811e-06	Neu1
PFN1	5.149761e-11	0.2071631	0.880	0.787	1.262155e-06	Neu1
SELL	5.172237e-11	0.1656462	0.942	0.873	1.267664e-06	Neu1
CTSD	1.757882e-37	0.63181224	0.583	0.386	4.308394e-33	Neu1
CTSH	9.975298e-13	0.28615973	0.072	0.024	2.444846e-08	Neu1

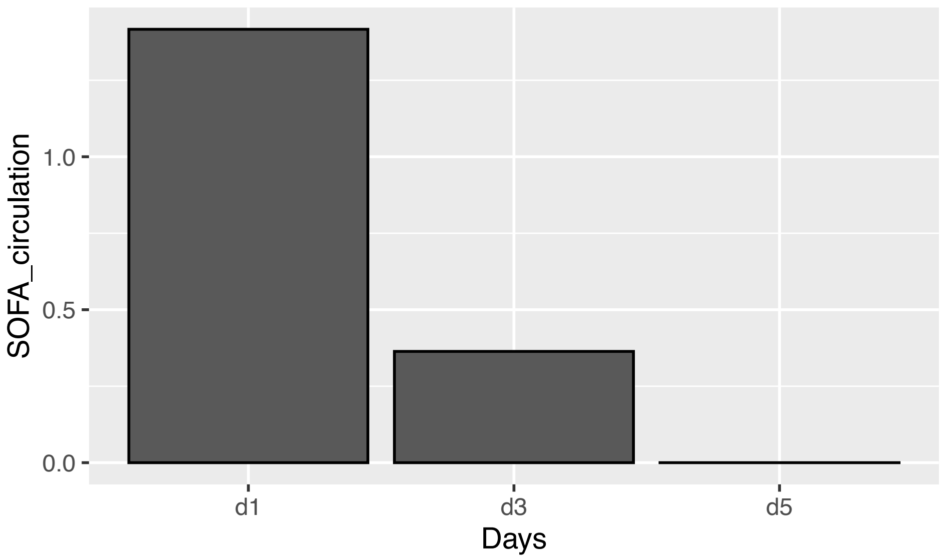

Figure S11. Changes of the mean $SOFA_{circulation}$ across sepsis days 1, 3 and 5, Related to Figure 5.

SOFA_circulation = the circulation component of the SOFA score.

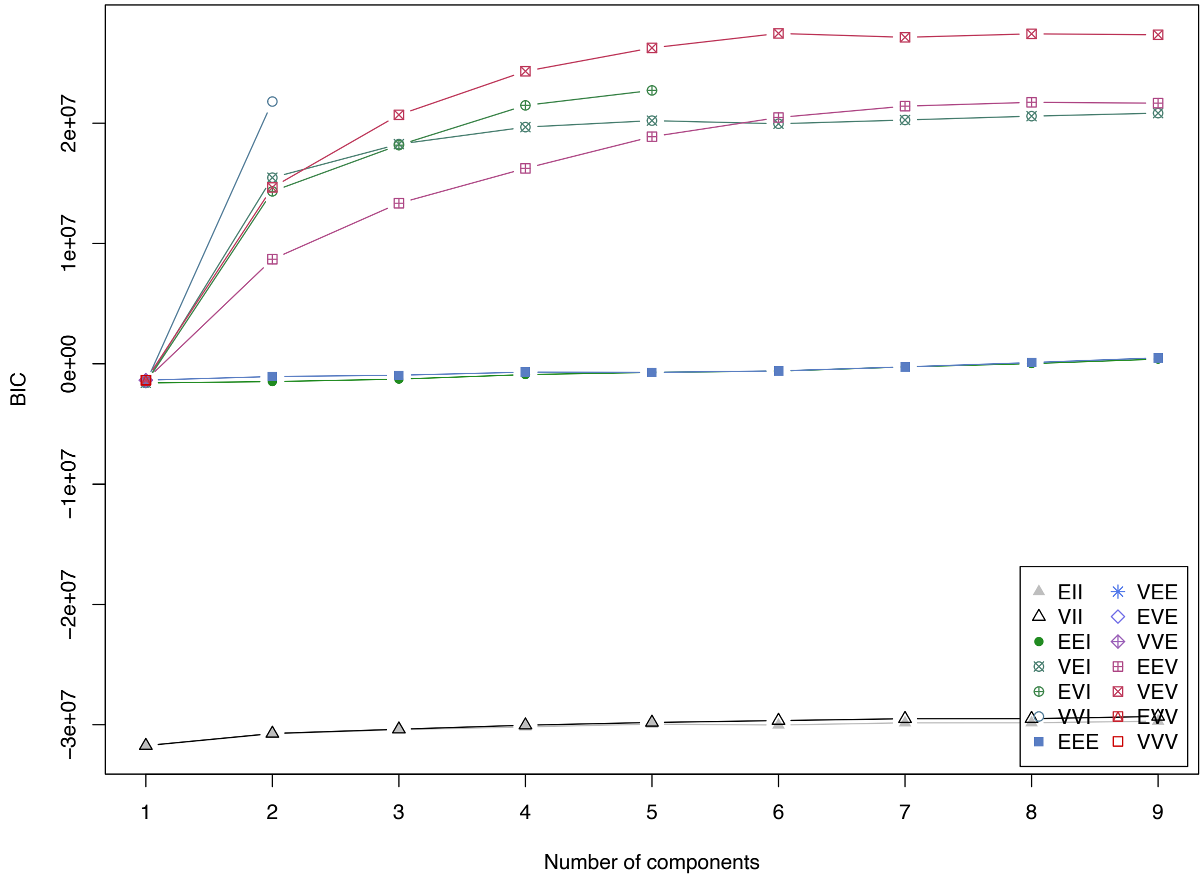

Figure S12. The best number of components for the gaussian mixture model were chosen according to the BIC values across the number of components, Related to Figure 5.

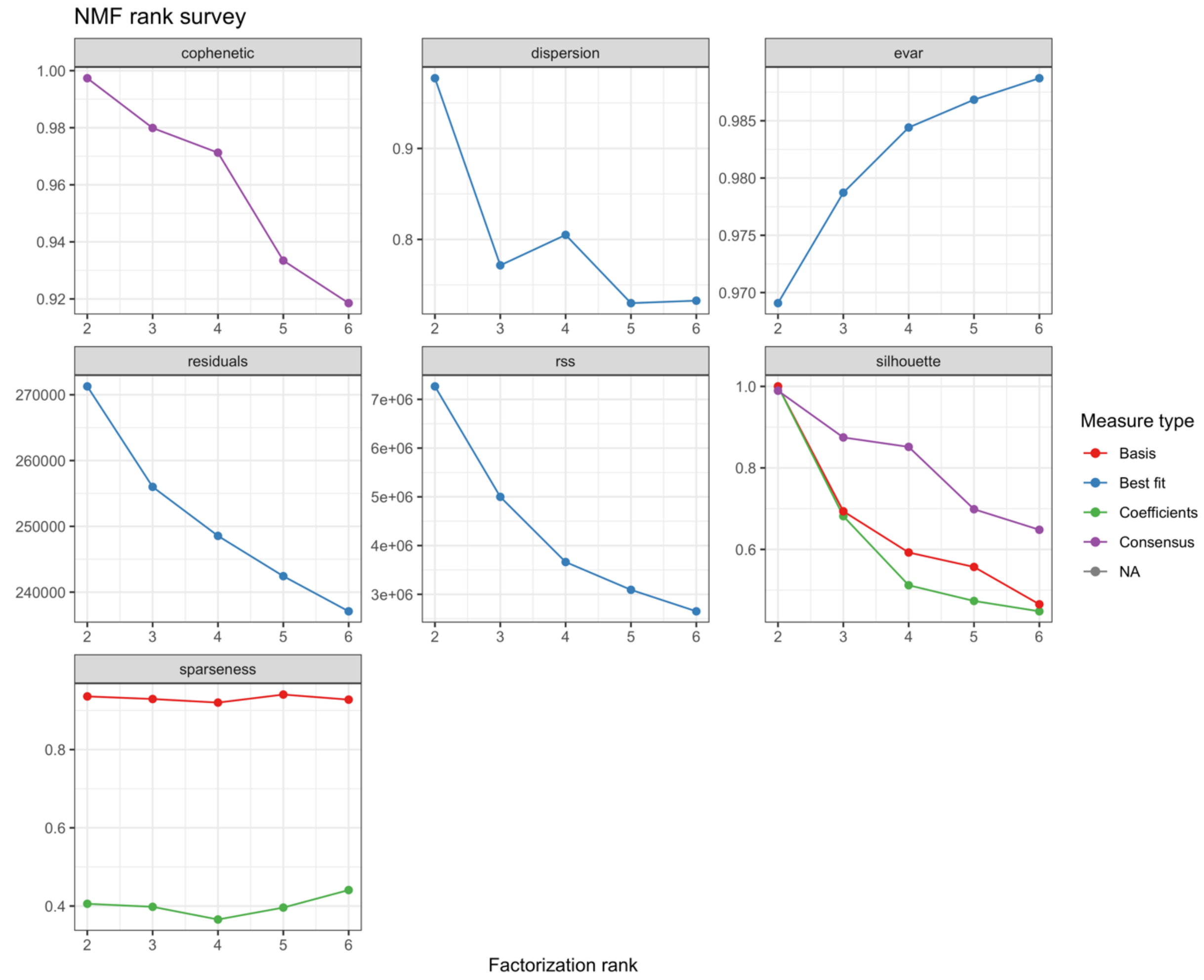

Figure S13. The best number of ranks for the non-negative matrix factorization, Related to Figure 5.

Four modules were considered to best describe the gene expression profile in Neu1.

Table S3. Characteristics of external datasets used for external validation, Related to Figure 6.

GEO accession	First author + year	Study design	Setting	Sample size	RNA quantification	Comparison groups
GSE74224	McHugh 2015	Observational, non-interventional study	tertiary ICUs	105	Microarrays	Post-surgical vs. sepsis
GSE134347	Scicluna 2020	prospective observational study	mixed ICUs of two tertiary teaching hospitals	156	next-generation microarray analysis	healthy subjects vs. critically-ill patients with sepsis vs. non-infectious conditions
GSE131761	Martínez-Paz 2021	patients following surgery recruited sequentially	Adult ICU	133	microarray analysis	Non-shock vs. septic shock

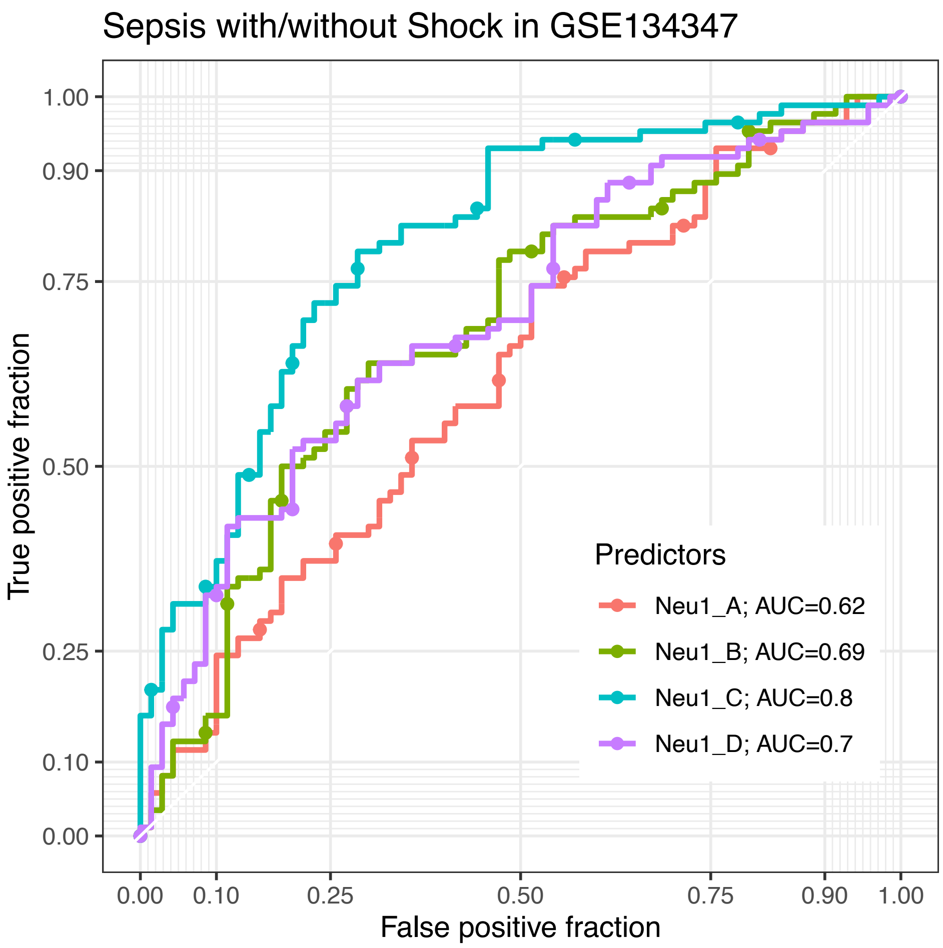

Figure S14. External validation of the Neu1 gene expression modules in differentiating shock versus non-shock, Related to Figure 6.
